# Extracellular Vesicles from Human Induced Pluripotent Stem Cells Exhibit a Unique MicroRNA and CircRNA Signature

**DOI:** 10.7150/ijbs.100113

**Published:** 2024-11-22

**Authors:** Mario Barilani, Valeria Peli, Paolo Manzini, Clelia Pistoni, Francesco Rusconi, Eva Maria Pinatel, Francesca Pischiutta, Dorian Tace, Maria Chiara Iachini, Noemi Elia, Francesca Tribuzio, Federica Banfi, Alessandro Sessa, Alessandro Cherubini, Vincenza Dolo, Valentina Bollati, Luisa Fiandra, Elena Longhi, Elisa R Zanier, Lorenza Lazzari

**Affiliations:** 1 Unit of Cell and Gene Therapies, Fondazione IRCCS Ca' Granda Ospedale Maggiore Policlinico, Milano, Italy.; 2 Department of Medical Oncology and Hematology, University Hospital Zurich, Switzerland.; 3 ITB-CNR, Institute of Biomedical Technologies, National Research Council, Segrate, Italy.; 4 Laboratory of Traumatic Brain Injury and Neuroprotection, Department of Acute Brain and Cardiovascular Injury, Istituto di Ricerche Farmacologiche Mario Negri IRCCS, Milano, Italy.; 5 San Raffaele Scientific Institute, Division of Neuroscience, Neuroepigenetics Unit, Milano, Italy.; 6 Department of Life, Health and Environmental Sciences, University of L'Aquila, L'Aquila, Italy.; 7 EPIGET Lab, Department of Clinical Sciences and Community Health, University of Milan, Milano, Italy.; 8 Department of Biotechnology and Biosciences, University of Milan-Bicocca, Milano, Italy.; 9 Laboratory of Transplant Immunology SC Trapianti Lombardia - NITp. Fondazione IRCCS Ca' Granda Ospedale Maggiore Policlinico, Milano, Italy.

**Keywords:** extracellular vesicles, exosomes, nanoparticles, human-induced pluripotent stem cells, miRNA, circRNA, cord blood

## Abstract

Extracellular vesicles (EV) have emerged as promising cell-free therapeutics in regenerative medicine. However, translating primary cell line-derived EV to clinical applications requires large-scale manufacturing and several challenges, such as replicative senescence, donor heterogeneity, and genetic instability.

To address these limitations, we used a reprogramming approach to generate human induced pluripotent stem cells (hiPSC) from the young source of cord blood mesenchymal stem/stromal cells (CBMSC). Capitalizing on their inexhaustible supply potential, hiPSC offer an attractive EV reservoir.

Our approach encompassed an exhaustive characterization of hiPSC-EV, aligning with the rigorous MISEV2023 guidelines. Analyses demonstrated physical features compatible with small EV (sEV) and established their identity and purity. Moreover, the sEV-shuttled non-coding (nc) RNA landscape, focusing on the microRNA and circular RNA cargo, completed the molecular signature. The kinetics of the hiPSC-sEV release and cell internalization assays unveiled robust EV production and consistent uptake by human neurons. Furthermore, hiPSC-sEV demonstrated *ex vivo* cell tissue-protective properties. Finally, via bioinformatics, the potential involvement of the ncRNA cargo in the hiPSC-sEV biological effects was explored.

This study significantly advances the understanding of pluripotent stem cell-derived EV. We propose cord blood MSC-derived hiPSC as a promising source for potentially therapeutic sEV.

## Background

Extracellular vesicles (EV) are nanometer-sized lipid bilayer membrane-bound structures that contain bioactive molecules, including nucleic acids, proteins, and lipids. EV trafficking serves as a fundamental mechanism for intercellular communication exchange 1. In view of possible clinical applications based on EV, it has become evident that utilizing the EV in therapy can offer several advantages compared to using parental cells (2): i) EV can be isolated and stored long-term at low temperatures, eliminating the need to produce large amounts of cells at the time of inoculation, which is required for cellular therapy 2-4; ii) EV contents are encapsulated and protected from degradation *in vivo* 5,6; iii) EV are stable bioactive entities 7,8; iv) EV are able to reach distant targets via blood circulation, as demonstrated by their intravenous administration in primates (10); and v) EV present reduced risks of undesired side effects compared to whole cells, particularly because EV are hypo/non-immunogenic, and therefore, rarely are able to induce immune rejection 9-11.

Despite the considerable progress in the EV research field and the advantages of cell-free therapy over cell therapy, the evaluation of EV in regenerative medicine approaches deals with challenges in achieving clinical applications 12,13. Among them, one major issue derives from cell identity and their culture conditions, which affect EV properties. Obtaining EV from cultured primary cell lines, such as mesenchymal stromal cells (MSC) often used in regenerative medicine, raises concerns regarding widespread heterogeneous isolation and cell culture methodologies, limited replication potential, establishment of senescence 14,15, genetic instability during prolonged cell expansion 16,17, and heterogeneity within and among cell donors 18,19. A multitude of such variables makes it difficult to define the EV characteristics (molecular identity, functionality, quality, and purity) that are crucial for obtaining consistent functional results, which are essential for the clinical translation of a potentially therapeutic EV product 13,20,21.

To overcome the lifespan limitations of any primary cell lines as an EV source for therapy, some researchers have implemented immortalization techniques 22. In the present work, an alternative possibility was explored generating a source of EV by reprogramming a fetal source-derived MSC 23 to human induced pluripotent stem cells (hiPSC) 24, utilizing a non-integrative and current good manufacturing practice (cGMP)-compliant method 25,26. By employing Sendai virus, we avoided tumorigenic risks associated with immortalization techniques, insertional mutagenesis, forced expression of oncogenes, genomic modification, and instability 27. Therefore, we investigated the physical and biological features of hiPSC-derived EV, following the MISEV2023 guidelines 28, and we demonstrated their tissue protective properties. Additionally, we explored the biological roles of the EV-shuttled circular (circ)RNAs and their potential micro (mi)RNA targets. Based on our findings, we propose that cord blood MSC-derived hiPSC serve as an optimal young stem cell source for potentially therapeutic EV. Overall, this study sheds light on the promising applications of hiPSC-EV in regenerative medicine and highlights their potential to go beyond current limitations in EV-based therapies.

## Methods

### Culture of human induced pluripotent stem cells

hiPSC (n=3) were generated from cord blood MSC and characterized following the previously described procedures 24. The hiPSC cultures were maintained in StemMACS iPS-Brew XF PSC medium (Miltenyi Biotec, Bergisch Gladbach, Germany). Upon reaching 80% confluence, the colonies were detached in accordance with the respective experimental requirements. For standard hiPSC culture maintenance, cells were incubated with 5 mM ethylenediaminetetraacetic acid disodium salt (EDTA; Sigma-Aldrich, St. Louis, Missouri, USA) in D-PBS without Ca^2+^ or Mg^2+^ (Euroclone, Milan, Italy) and seeded as cell clumps on hESC-qualified Matrigel Matrix-coated culture plates (Corning, Corning, New York, USA). For EV production and kinetics, cells were incubated with Accutase (Biowest, Nuaillé, France) and seeded as single cells in the presence of Y-27632 RHO/ROCK pathway inhibitor (Stem Cell Technologies, Vancouver, Canada) at a density of 5,000 cells/cm^2^ on Truncated Vitronectin Recombinant Human Protein-coated culture surfaces (VTN-N; Thermo Fisher Scientific, Waltham, Massachusetts, USA). hiPSC identity was confirmed using short tandem repeat (STR) profiling (data not shown).

### Nanoparticle tracking analysis

Nanoparticle Tracking Analysis (NTA) was performed using a NanoSight NS300 (Malvern, Surrey, UK). Samples were diluted with 0.1 μm tri-filtered D-PBS (Euroclone) to optimize the quality parameters for analysis. Media incubated for 24 h at 37 °C in cell-free wells of the plates were used as blank. The diluted samples were analyzed using a low-volume flow-cell chamber in flow mode, with 5 recordings of 60 s each to ensure a constant sample flow.

### Isolation of extracellular vesicles

For EV isolation, cell culture supernatants were collected on two consecutive days at 70-80% confluence. Cell supernatants were processed through serial centrifugation as previously described 29, with minor modifications. Briefly, cell culture supernatants were pooled, centrifuged at 350 ×g for 10 min at room temperature (21-25 ºC, RT), collected, and further centrifuged at 4,700 ×g for 15 min at RT. The resulting cleared supernatants were 0.2 μm filter-sterilized and ultracentrifuged at 100,000 ×g for 1 h at 4 °C using a Sorvall WX 80+ ultracentrifuge equipped with F37L-8×100 Fiberlite fixed angle rotor (Thermo Fisher Scientific). The EV-containing pellets were resuspended and successively washed with 0.1 μm tri-filtered D-PBS. The supernatant was discarded, and the obtained ultracentrifuged small EV pellet (hiPSC-sEV) was resuspended in a total volume of 200 μL. When specified, EV contained in the cleared supernatant were concentrated by ultrafiltration (UF-EV) at 4,000 ×g using 30 kDa Amicon Ultra-15 tubes (Merck, Darmstadt, Germany).

### Electron microscopy

For Transmission Electron Microscopy (TEM), hiPSC-sEV were prepared as previously described and analyzed within 24 h. The sample was adsorbed onto 300 mesh carbon-coated copper grids (Electron Microscopy Sciences, Hatfield, Pennsylvania, USA) and fixed with 2% glutaraldehyde in D-PBS. Grids with adhered hiPSC-sEV were examined using a Philips CM 100 TEM microscope (Philips, Amsterdam, Netherlands.) at 80 kV after negative staining with 2% phosphotungstic acid (Sigma-Aldrich) and images were captured using a digital camera (Kodak, Rochester, New York, USA; or using Jeol JEM 2100Plus (Jeol, Tokyo, Japan) electron microscope equipped with a 9 MP complementary metal oxide superconductor (CMOS) and Gatan Rio9 digital camera (Gatan, Inc. Pleasanton, CA, USA).

### Sucrose density gradient

Tubes containing linear sucrose density gradients were manually prepared. A volume of 2.5 mL of 2.0 M, 1.4 M, 0.8 M, and 0.25 M sucrose (Merck) and 20 mM HEPES (Sigma-Aldrich) MilliQ water solutions were sequentially pipetted into an open-top polyclear centrifuge tube (Seton, USA). EV-containing pooled size-exclusion chromatography (SEC) fractions were loaded onto the gradient and ultracentrifuged 200,000 ×g overnight at 4 °C on a Sorvall WX 80+ ultracentrifuge (Thermo Fisher Scientific) equipped with TH-641 Swinging Bucket Rotor (Thermo Fisher Scientific). Eight fractions were collected, and the refractive index of each fraction was measured using a HI96800 refractometer (Hanna Instruments, Woonsocket, Rhode Island, USA) with sucrose temperature compensation (nD20). Each fraction was then washed through ultracentrifugation with a F37L-8×100 Fiberlite fixed angle rotor (Thermo Fisher Scientific) and resuspended in a total volume of 100 μL of 0.1 μm tri-filtered D-PBS for further analysis.

### MACSPlex assay

The Human MACSPlex Exosome Kit (Miltenyi Biotec) was used in the cleared supernatants as previously described 29. Analysis and data processing were performed on a FACSCanto II cytometer using BD FACSDiva software (BD, Franklin Lakes, New Jersey, USA).

### Western blotting

Proteins were extracted in RIPA buffer (Sigma-Aldrich), following standard procedures. The protein concentration was determined using the Pierce BCA Protein Assay Kit (Thermo Fisher Scientific), according with manufacturer's instructions. To evaluate tetraspanin levels (CD63, CD9, and CD81), 40 μg of proteins were separated on Novex WedgeWell 4-20% Tris-Glycine Gels (Thermo Fisher Scientific) under non-reducing conditions. To detect other proteins, 8-40 μg of proteins were separated on a 10% polyacrylamide gel (Sigma-Aldrich) under reducing conditions with a 10X Bolt Sample Reducing Agent (Thermo Fisher Scientific). Samples were boiled at 95 °C for 5 min, using 4X NuPAGE LDS Sample Buffer (Thermo Fisher Scientific). All gels were blotted using an iBlot 2 Gel Transfer Device (Thermo Fisher Scientific) with iBlot PVDF or nitrocellulose Transfer Stacks (Thermo Fisher Scientific), as specified in Table 1. Membranes were blocked with 5% nonfat milk and incubated with the respective primary antibodies overnight at 4 °C, then with the appropriate secondary antibody. The antibodies used are listed in Tables 1 and 2. The proteins of interest were visualized using the Amersham ECL Prime Western Blotting System (GE Healthcare, Chicago, Illinois, USA). Chemiluminescence images were obtained using ChemiDoc XRS+ (Bio-Rad, Hercules, California, USA).

### Size-exclusion chromatography

SEC was performed according to a modified version of a previously described protocol 30. SEC columns were prepared in 10 mL plastic syringes: the tip of the syringe was filled with a nylon stocking filter and 10 mL of Sepharose (Sigma-Aldrich) was poured into the syringe to form a 1.5 cm-diameter and 4.5 cm-height column. hiPSC-sEV or hiPSC-UF-EV samples were resuspended in 0.1 μm tri-filtered D-PBS and loaded on the column. For the Carboxyfluorescein succinimidyl ester (CFSE)-labelling protocol, elution was performed using 0.1 μm tri-filtered Neurobasal (Gibco). Twenty sequential fractions (0.5 mL) were collected and processed immediately or within 24h for further analysis.

### miRNome PCR-array

The miRNome of hiPSC-sEV was extracted using the miRNeasy Mini Kit (Qiagen, Venlo, Netherlands) and the RNeasy MinElute Cleanup Kit (Qiagen), following the manufacturer's instructions. The extracted RNA was retrotranscribed using the TaqMan Advanced miRNA cDNA Synthesis Kit (Thermo Fisher Scientific), and analyzed using the TaqMan OpenArray Real-Time PCR Master Mix and TaqMan OpenArray Human MicroRNA Panel array (Thermo Fisher Scientific) on a QuantStudio 12 K Flex Real Time PCR System (Thermo Fisher Scientific) 24. Dead entries based on the current miRBase version 22.1 database were excluded for further analysis.

### circRNA micro-array

The extracted total RNA was enriched in circRNAs using a RNase R treatment (Epicentre Biotechnologies, Madison, WI, USA). The RNA samples were amplified and transcribed into fluorescent cRNA using the Super RNA Labeling Kit random priming method (ArrayStar, Carlsbad, California, USA). Hybridization was performed using an Arraystar Human Circular RNA Microarray (Arraystar V1.0). Scanning was performed using an Agilent Scanner G2505C, and raw data were extracted using the Agilent Feature Extraction software (version 11.0.1.1). The identification of circRNAs followed the circBASE database nomenclature. A quality threshold of the 90^th^ percentile was applied to the signal intensity to retrieve a list of the most abundant molecules 31. Results were archived in the NCBI GEO database under the series accession number GSE240004. Comparison with hiPSC was performed using a previously published dataset available in the NCBI GEO database under the series accession number GSE144629.

### Neural progenitor cell-derived postmitotic neurons differentiation

Neural progenitor cells (NPCs) were generated from fibroblast-derived hiPSC 32 and cultured onto matrigel-coated flasks in NPC medium containing DMEM/F12, N-2 and B-27 supplements (Thermo Fisher Scientific), 1% Pen/Strept, and 20 ng/ml bFGF (Thermo Fisher Scientific). NPCs were passaged twice a week using Accutase solution (Sigma-Aldrich).

For neurons differentiation, medium of 90% confluent NPCs was replaced with differentiation medium composed of DMEM/F12, N-2 and B-27 supplements (Thermo Fisher Scientific), 1% Pen/Strept, 10 μM SU5402 (Sigma-Aldrich,), 8 μM PD0325901 (Sigma-Aldrich), 10 μM DAPT (Sigma-Aldrich). Differentiation medium was replaced every day with a fresh one on days 1 and 2. At day 3, cells were detached with Accutase (Sigma-Aldrich) and seeded at a density of 75,000 cells/cm^2^ onto poly-L-lysine/laminin/fibronectin (100 μg/ml, 2 μg/ml, 2 μg/ml) (Sigma-Aldrich)-coated coverslip in neuronal maturation medium supplemented with 10 μM ROCK inhibitor Y27632 for the first 24 h.

Neuronal maturation medium was composed by Neurobasal A (ThermoFisher Scientific) supplemented with 1× B-27 supplement, 2 mM glutamine, 1% Pen/Strept, 20 ng/ml BDNF (Peprotech), 100 nM ascorbic acid (Sigma-Aldrich), 1 μg/μl Laminin (Sigma- Aldrich), 10 μM DAPT (Sigma- Aldrich), 250 μM dbcAMP (Selleckchem). The culture medium was replaced the next day to remove the ROCK inhibitor; then half of the medium was replaced with a fresh neuronal maturation medium twice a week.

### Extracellular vesicles labeling

hiPSC-sEV were mixed in Diluent C (Sigma-Aldrich) and PKH26 (Sigma-Aldrich) and incubated for 20 minutes at RT in the dark. The reaction was stopped by adding an equal volume of 1% Bovine Serum Albumin (BSA) (Sigma Aldrich). hiPSC-sEV were then ultracentrifuged at 100,000 xg for 1 hour and resuspended in D-PBS (Euroclone). For CFSE labeling, CellTrace CFSE Cell Proliferation Kit (Thermo Fisher Scientific) was used at a final concentration of 20 µM to stain hiPSC-sEV preparations containing 1.2-2.4 × 10^12^ particles/mL. After incubation for 2 h, the hiPSC-sEV were washed through ultracentrifugation and further purified by SEC. Fractions 6 and 7 were collected and pooled for subsequent use.

### Flow cytometry

To evaluate CFSE+ hiPSC-sEV, a specific setup for nanoscale flow cytometry was implemented on a FACSCanto II cytometer using FACSDiva software (BD). At least 1,000 events were acquired within P1 gate at a low acquisition flow rate. The acquired particles were plotted against SSC-H and FL1-H to determine the percentages of CFSE-positive events. Megamix-Plus SSC polystyrene beads (160, 200, 240, and 500 nm) (Stago, Asnières-sur-Seine, France) were used for quality control following the manufacturer's instructions. Standard flow cytometry was performed to evaluate hiPSC-sEV uptake by neurons.

### Immunofluorescence staining and acquisition protocol

Neurons (75,000 cells/cm^2^) were incubated for 24h with 10^6^ particles (PKH26-hiPSC-sEV) per cell 33 and then analyzed by confocal microscopy.

Samples were then fixed in 4% paraformaldehyde for 20 min on ice, washed and permeabilized for 30 min 0,3% Triton (Eurobio Scientific, Les Ulis, France), 3% BSA (SERVA Electrophoresis GmbH, Heidelberg, Germany). Then, cells were incubated with chicken polyclonal anti-human MAP2 primary antibody 1:1000 (ab92434, abcam) overnight at 4°C; the day after, with goat anti-chicken secondary antibody 1:1000 (AlexaFluor-647, Thermo Fisher Scientific) for 1h at RT and with 0.1 μg/mL DAPI (Roche, Basel, Switzerland). Glass dishes were mounted on ProLong Gold Antifade Mountant (Thermo Fisher Scientific).

Immunofluorescence imaging was performed using a Leica SP8 Stellaris confocal microscope (Leica, Wetzlar, Germany), managed by LASX software. The acquisition was taken with a white light laser and Diode 405, using the HC PL APO CS 2 63X/1.30 GLYC NA objective. Each ROI was 2048 x 2048, zoom 1.28, with a pixel size of 0.071µm and a voxel size of 0.071µm (acquired at 400 Hz). For the orthogonal views, images were acquired with the same objective, and were 2768 x 2768, zoom 1.28, having a pixel size of 0.052 µm and a voxel size of 0.052µm; 15 steps of 0.633 µm (acquired at 428Hz).

### *Ex vivo* model of brain ischemia

Organotypic cortical brain slice preparation was performed as previously described 34, starting from the prefrontal cortex of C57BL/6 mouse pups (P1-3). After one week in culture (day 0), cortical slices were subjected to oxygen and glucose deprivation (OGD), using an hypoxic chamber (Whitley H35 Hypoxystation, Don Whitley Scientific, UK) at 37 ºC, [O_2_]=0.1%, [CO_2_]=5%, [N_2_]=95% for 2 h in deoxygenated glucose-free medium. One hour after OGD, cortical slices were treated with different doses of hiPSC-sEV (0.6-6-60 × 10^9^ particles/well/administration, named 1x, 10x, 100x) delivered in the culture medium. At 24h, the culture medium was changed and freshly sEV were administered at the same concentration. The collected medium was analysed for neurofilament light chain (NfL) release. Forty-eight hours after OGD, organotypic slices were analyzed for cell death using a propidium iodide incorporation assay. Slices were collected using the TRIzol reagent (Thermo Fisher Scientific) for subsequent gene expression studies.

### Propidium iodide incorporation

To evaluate cell death 48 h after OGD injury, the inserts with cortical slices were placed on new plates with fresh NB/B27 medium (Invitrogen, Waltham, Massachusetts, USA) containing 2 µM of propidium iodide (PI; Sigma-Aldrich, USA) 35 and incubated for 30 min. Images were acquired using the TRITC filter of an Olympus IX71 microscope at X4 magnification (Olympus, Tokyo, Japan) and analyzed using Fiji software (University of Wisconsin-Madison, USA). Fluorescence intensity per slice was measured as Integrated Density and the value was normalized over the slice area (in mm^2^).

### Quantification of neuronal injury biomarker in the culture medium

To assess neuronal damage, the amount of neurofilament light chain (NfL) released in the culture media, collected and stored at -20°C, was quantified. Analysis was performed using a commercially available single molecule array (simoa) immunoassay (Quanterix, Billerica, MA, USA) on an SR-X Analyzer (Simoa® NF-light™ V2 Advantage Kit, Item 104073) as described by the manufacturer. A single lot of reagents was used for all samples.

### qPCR (quantitative Polymerase Chain Reaction)

A CFX96 Real-Time System coupled with a C1000 Thermal Cycler (Bio-Rad) was used for all qPCR experiments. Data were analyzed and exported for analysis using the CFX Manager software (Bio-Rad). For miRNA validation, miRNA-enriched RNA was extracted from hiPSC-sEV or SEC fractions, as described above. Retrotranscription was performed using a miScript II RT Kit (Qiagen) or by cDNA Reverse Transcription (RT) kit (Applied Biosystems). Real-time PCR was performed using miScript SYBR Green PCR Kit (Qiagen) and miScript Primer Assays (Qiagen) for amplification of specific targets. Global normalization was performed, and the normalizing factor was calculated as the mean of 2^-ΔCt^ values of all genes analyzed.

For circRNA analysis, total RNA was extracted from hiPSC and hiPSC-sEV pellets using TRIzol reagent (Thermo Fisher Scientific). RNase R treatment (Epicentre Biotechnologies, Madison, WI, USA) was performed 26 prior to retrotranscription using SuperScript IV VILO Master Mix (Thermo Fisher Scientific), amplification using PowerUp SYBR Green Master Mix (Thermo Fisher Scientific), and global normalization. For gene expression analysis, the ΔΔC_t_ method was applied, using *Gapdh* as a housekeeping gene 36. For assessment of full-length mRNA, 250 ng RNA was retrotranscribed with SuperScript IV VILO Master Mix (Invitrogen) and amplified with DreamTaq PCR Master Mix (2X) (Thermo Fisher Scientific). Amplicons were detected by standard gel electrophoresis. The sequences of the designed primers or product codes of commercially available assays (Qiagen) are listed in Table 3.

### EV circRNA biological role prediction

To explore the potential involvement of the hiPSC-sEV circRNA cargo in the pathways regulated in the organotypic cortical brain slice OGD model, the 10 most expressed circRNAs were selected based on their normalized array signals. Their potential miRNA targets were predicted using TargetScan, PITA, and miRanda algorithms 37-39 requiring specific parameters for prediction: miRanda score over 80 and energy lower than 15, and for PITA, dGduplex_miRNA lower than -15 and dGopen_miRNA higher than -15. The list of miRNAs with a minimum of two binding sites (on the same or on different circRNAs), according to the predictions of at least two algorithms, was ordered based on their frontal lobe expression signal in the miRNA tissue Atlas v2.0 40 to obtain a list of 15 miRNAs most probably targeted in our biological context. To predict their biological roles, the multiMiR R package 41 was employed to retrieve miRNA-validated targets, filtering the most consistent results (only PAR-CLIP|HITSCLIP|CLASH|Luciferase|Degradome|ChIP-seq|ELISA|Immuno. Supporting data were selected after excluding weak MTI findings). The DOSE package 42 was used to calculate enrichment in the DISgeNet database 43 while clusterProfiler was adopted for gene ontology (GO) biological process enrichments. The results were then manually refined to better contextualize them in our biological context, focusing on ischemic and hypoxia-related brain diseases and hypoxia, ischemia, apoptosis, cell death, and cytokine-related terms among the biological processes. Only the terms with adjusted p-values lower than 0.05 were considered as enriched. The *enrichplot* functions were used to graphically represent the results.

### Reference databases and statistical analysis

The miRNA and circRNA data were annotated and analyzed using various reference databases and software tools. The miRbase 22.1 database was utilized for miRNA nomenclature and identification (https://mirbase.org/) 44. The HGNC Database, HUGO Gene Nomenclature Committee (HGNC), European Molecular Biology Laboratory, European Bioinformatics Institute (EMBL-EBI), Wellcome Genome Campus, Hinxton, Cambridge CB10 1SD, United Kingdom, was employed to study miRNA families and clusters (https://www.genenames.org) 45. For experimentally validated miRNA-target interactions, the miRTarBase 9.0 beta (https://mirtarbase.cuhk.edu.cn) 46 was thoroughly investigated. For the circRNA study, the CircBase from the July 2017 update (http://www.circbase.org/) 47 was used as a reference. To create visual representations of the miRNome heatmap and circRNA plots, we employed the gplots package and heatmap.2() function in R (R Core Team (2018). R: Language and environment for statistical computing. R Foundation for Statistical Computing, Vienna, Austria. Available online at https://www.R-project.org.

For miRNA profile comparison, top expressed miRNA lists were retrieved from tables or supplementary materials reported by other groups 48-50 and limited to the first 20 entries if longer. MiRNA names were updated to mirBase version 22.1, if needed, to compare common entries. Venn diagram representation of the miRNA common to one or more over the four examined profiles was produced online by Venny (Oliveros, J.C. (2007-2015) Venny. An interactive tool for comparing lists with Venn's diagrams. https://bioinfogp.cnb.csic.es/tools/venny/index.html).

All statistical analyses and graphical representations were performed using Prism 6 software (GraphPad Software, GraphPad, La Jolla, California, USA). Details of the specific statistical analysis methods are detailed in the Figure legends. Statistical significance was set at p < 0.05.

## Results

### Human induced pluripotent stem cells release small extracellular vesicles

**Figure 1:**
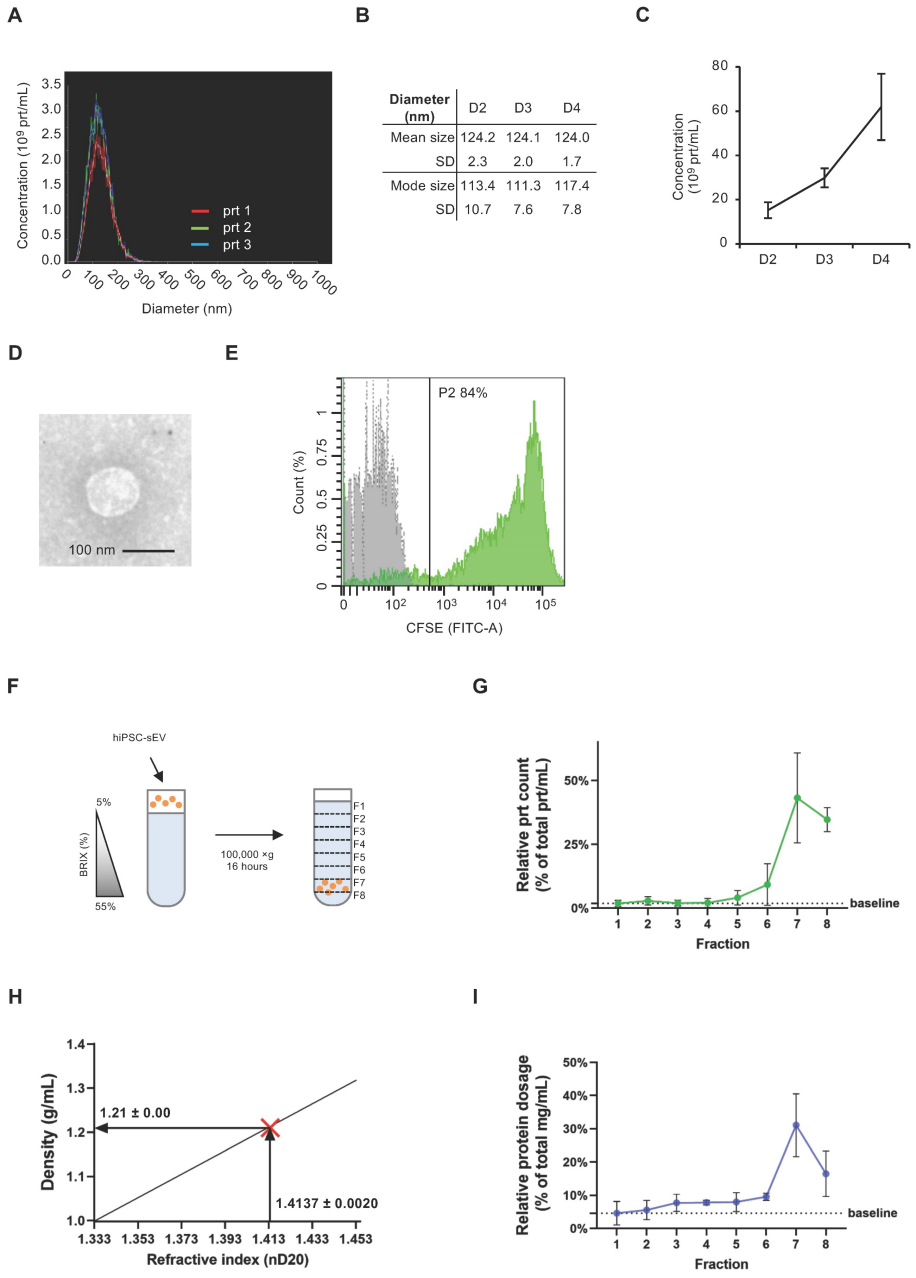
** Human induced pluripotent stem cells release small extracellular vesicles.** Overlayed histograms (A) show size distribution profile of particles released by hiPSC (n=3). Table (B) and scatter plot (C) represent mean, mode size and concentration of hiPSC particles (n=3) released during four days of culture (D2, D3, D4) as mean and standard deviation (SD). Representative TEM image (D) shows morphology and size of sEV released by hiPSC; scale bar is 100 nm. Histogram in (E) shows overlayed fluorescent signal from unlabeled (grey) and CFSE+ (green) hiPSC-sEV. Scheme (F) summarizes the protocol implemented for SDG separation of hiPSC-sEV. Plot in (G) reports the relative particle count calculated as percentage (%) of total prt/mL values for each fraction of the SDG; mean and SD are represented (n=3). Plot in (H) visually shows the estimation of hiPSC-sEV density starting from their refractive index, using the formula y=2.6564x-2.5421 (calculated based on standard conversion tables); hiPSC-sEV are indicated by the red X mark: vertical arrow pinpoints hiPSC-sEV refractive index, horizontal arrow pinpoints hiPSC-sEV density. Plot in (I) reports the relative protein dosage calculated as % of total protein concentration values for each fraction of the SDG; mean and SD are represented (n=3). Abbreviations: BRIX, sugar content of aqueous solution in percentage (%); D, days; F, fraction; hiPSC, human induced pluripotent stem cells; nD20, refractive index temperature compensated; P2, population 2 gate; prt, particles; SD, standard deviation; SDG, sucrose density gradient; TEM, transmission electron microscope; hiPSC-sEV, hiPSC-derived sEV.

The initial detection of EV release from hiPSC involved performing NTA on cleared supernatants from hiPSC cultures, diluted in D-PBS. This analysis revealed a nanoparticle population with a size distribution consistent with that of sEV (30) (Figure 1A). Notably, the 50^th^ percentile size was 125 ± 3 nm, while the 90^th^ percentile size was 185 ± 4 nm (Supplementary Figure 1A). Importantly, this dimensional profile remained consistent over successive days of hiPSC culture, as demonstrated by the mean and mode size values (Figure 1B). The same samples were analyzed using NTA to determine the kinetics of hiPSC-sEV release. The observed nanoparticle concentration per mL was approximately 15-60 x 10^9^, with a three-fold increase observed over three days of hiPSC culture (Figure 1C).

To validate the size and structure of the hiPSC-sEV, we concentrated them through ultracentrifugation and analyzed them using TEM. Images obtained corroborated the NTA findings, confirming the presence of EV with a diameter of 100 nm (Figure 1D).

We performed a CFSE assay to characterize the biological nature of hiPSC-sEV 28,51. The assay results indicated the integrity of these vesicles, with 79.0 ± 4.6% of CFSE-positive events, underscoring their status as membrane-enclosed bodies containing active enzymes (n=3) (Figure 1E).

To further confirm the vesicular identity of hiPSC-sEV, we employed a sucrose density gradient (SDG) to assess their flotation properties (Figure 1F). Following hiPSC-sEV separation, we collected eight fractions along the SDG (Supplementary Figure 1B). NTA analysis revealed a peak particle count in fraction 7 (Figure 1G), corresponding to a density of 1.21 ± 0.00 g/mL (Figure 1H). This observation was consistent with protein concentration peaks at fraction 7, as determined by the BCA assay, which is consistent with previous results (Figure 1I). Validation of hiPSC-sEV presence was obtained using TEM (Supplementary Figure 1C).

Altogether, our findings demonstrate that hiPSC release sEV with consistent physical and biological properties. Additionally, these results provide insights into optimal harvesting timing for hiPSC-sEV in subsequent studies.

### Extracellular vesicle protein cargo defines their identity and cell source

To elucidate the presence and relative abundance of markers associated with identity, cell type source, organelle origin, and biogenesis pathways, a comprehensive biochemical analysis was performed on hiPSC-sEV. This entailed surface antigen immunophenotyping and assessment of protein content.

**Figure 2:**
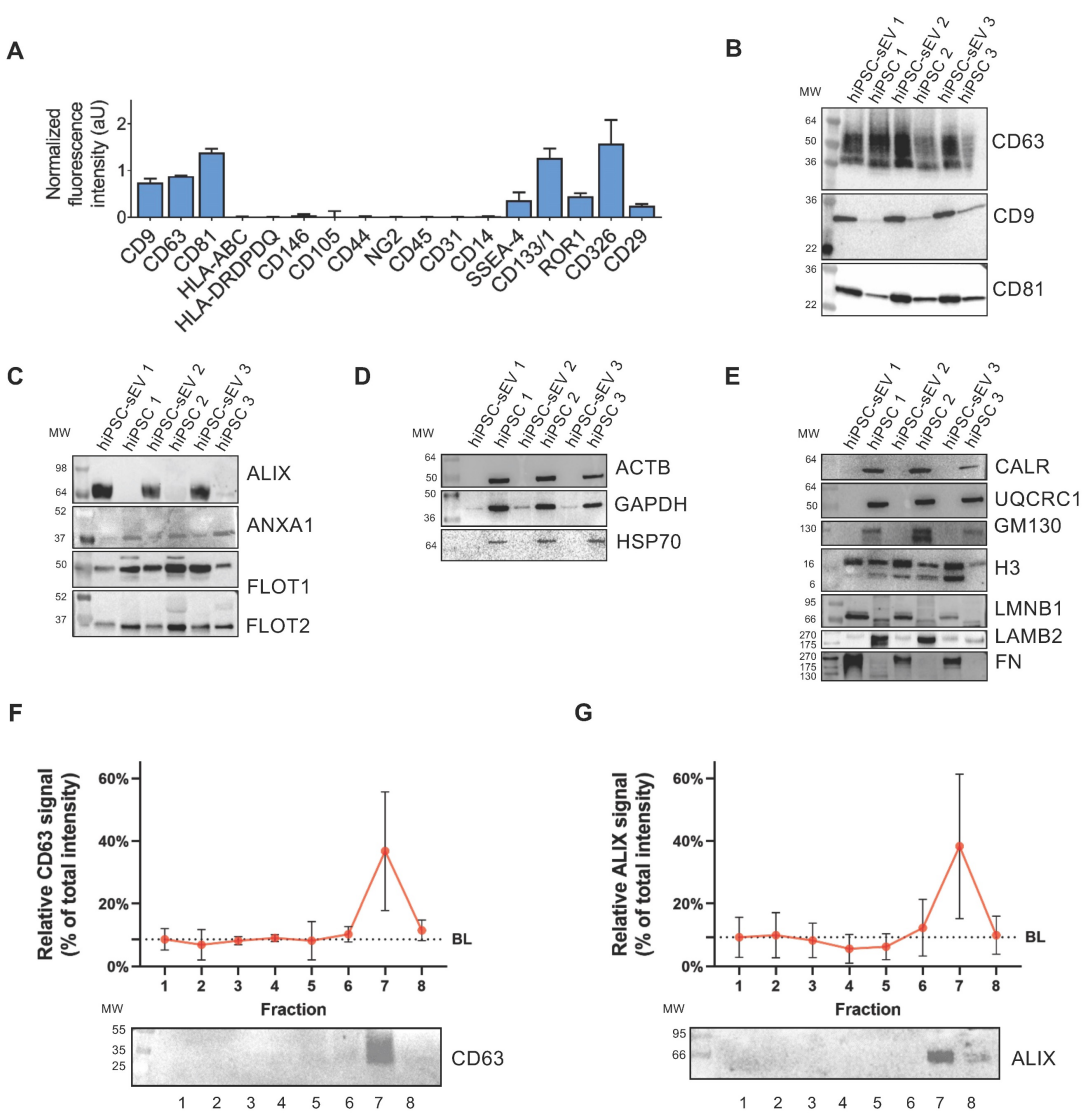
** hiPSC-derived extracellular vesicles are reminiscent of cell source and biogenesis pathway.** Histograms in (A) show signal intensity determined by flow cytometry, in arbitrary units of protein markers on the surface of sEV released by hiPSC; mean and standard deviation (SD) are represented (n=3). Blots in (B-E) show western analysis comparisons between parental hiPSC and released hiPSC-sEV (n=3) for surface and luminal protein markers. In particular: EV surface non-cell-specific markers (B), cytosolic EV-specific (C) and non-EV-specific markers (D), other intracellular compartments markers (E). Image (F): upper panel shows signal intensity distribution of a sEV protein surface marker as detected by western analysis after separation by SDG (n=3; mean and SD are represented); lower panel is a representative blot. Image (G): upper panel shows signal intensity distribution of a sEV protein luminal marker as detected by western analysis after separation by SDG (n=3; mean and SD are represented); lower panel is a representative blot. Abbreviations: AU: arbitrary units; BL: baseline; hiPSC: human induced pluripotent stem cells; MW: molecular weight; SDG: sucrose density gradient; hiPSC-sEV: hiPSC-derived small extracellular vesicles.

Utilizing a bead-based MACSplex assay in conjunction with flow cytometry, we detected the presence of EV-enriched tetraspanins CD9, CD63, and CD81. High levels of pluripotency/multipotent progenitor (SSEA-4, CD133/1), early embryonic (ROR1), and epithelial (CD326, CD29) cell markers were observed. Conversely, antigens linked to mesenchymal stromal cells (CD146, CD105, CD44, NG2) 29,52 and immune system cells (CD45, CD31, CD14) (26) were not detected (Figure 2A). Remarkably, major histocompatibility complex classes, HLA-ABC and HLA-DRDPDQ were not detected in hiPSC-sEV.

The tetraspanin content was further evaluated by western blot analysis, revealing hiPSC-sEV enrichment when compared to parental hiPSC (Figure 2B). Surface membrane antigens associated with pluripotency, TRA1-60, TRA1-81, and SSEA-4 were also detected, with higher abundance in hiPSC-sEV compared to hiPSC (Supplementary Figure 1D).

Examination of cytosolic proteins revealed that the EV marker ALIX (95 kDa) exhibited an exclusive signal in hiPSC-sEV, in contrast to other commonly used EV markers, ANXA1, FLOT1, and FLOT2 (39, 47, and 49 kDa, respectively), which were similarly represented in hiPSC or slightly more prominent than in released hiPSC-sEV (Figure 2C). Non-EV-specific cytosolic proteins ACTB, GAPDH, and HSP70 (42, 36, and 70 kDa, respectively) were exclusively or enriched in parental hiPSC, while they were faintly detected or absent in hiPSC-sEV (Figure 2D).

Further characterization was performed to exclude the presence of biological materials derived from other cellular compartments. Organelle markers for the endoplasmic reticulum (CALR, 46 kDa), mitochondria (UQCRC1, 53 kDa), and Golgi (GM130, 130 kDa) were detected only in hiPSC. Meanwhile, nuclear markers were detected in both parental hiPSC and hiPSC-sEV at similar levels (H3, 15 kDa), or enriched in hiPSC-sEV (LMNB1, 66-70 kDa). Secreted proteins and components of the extracellular matrix were either scarcely present (LAMB2, 220 kDa) or enriched (FN, 240 kDa) in hiPSC-sEV (Figure 2E).

Validation of widely accepted EV markers CD63 and ALIX was carried out in the context of sucrose density gradient (SDG) fractions. The results, consistent with NTA and BCA data, exhibited an exclusive signal for CD63 (Figure 2F) and a highly enriched signal for ALIX (Figure 2G).

These findings collectively demonstrate the presence of markers typifying EV and their biogenesis pathways within hiPSC-sEV. The presence of surface antigens characteristic of parental cells and the absence of antigens associated with other potentially co-isolating organelles align with the MISEV2023 guidelines 28.

### Size-exclusion chromatography reveals identity of pure extracellular vesicles

**Figure 3:**
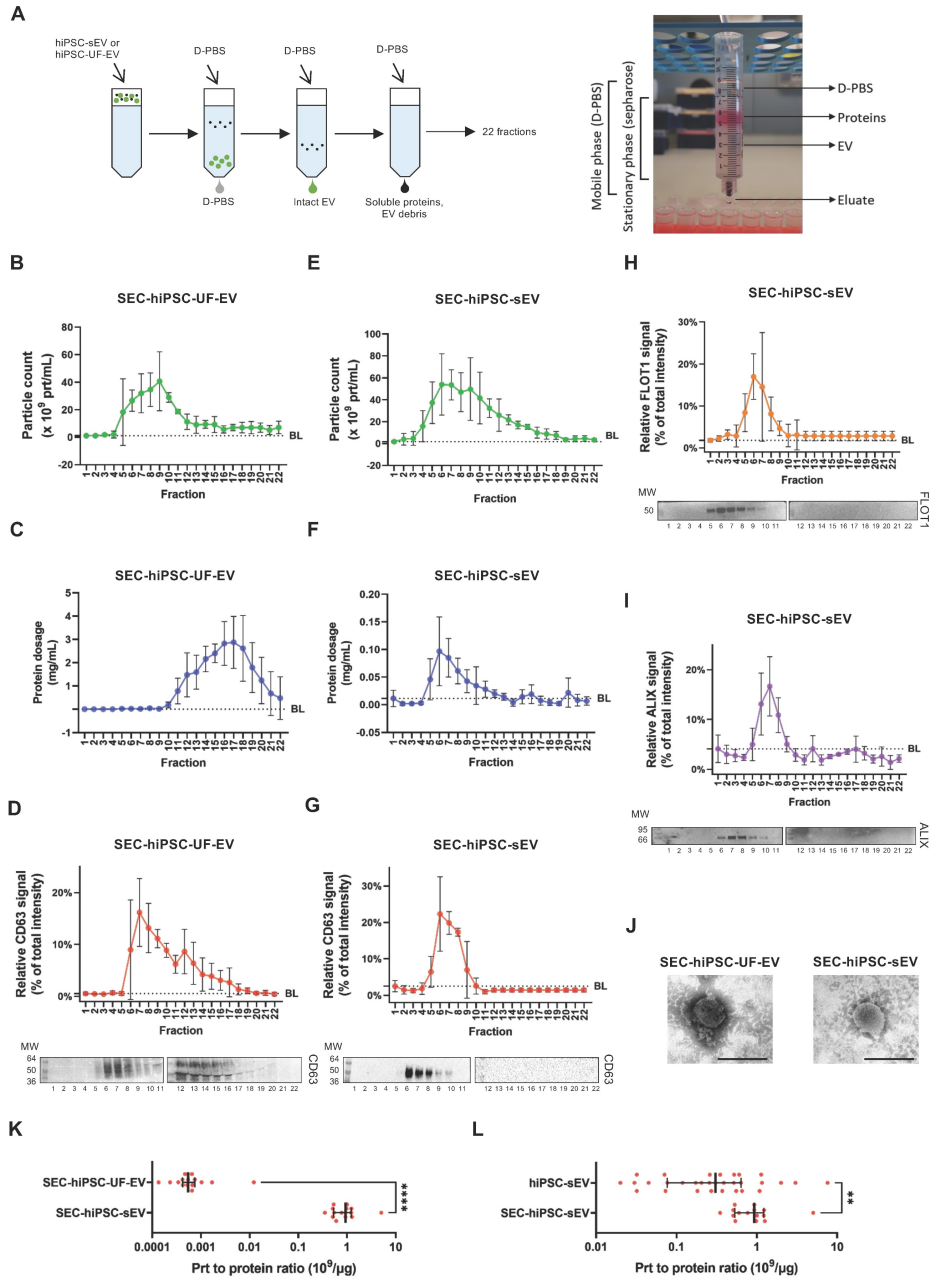
** Size-exclusion chromatography pinpoints integrity of pure hiPSC-derived extracellular vesicles.** Scheme in (A) summarizes the protocol implemented for SEC together with a photo of the home-made SEC system implemented. Plots show distribution of particle count (B), protein dosage (C) and protein surface marker signal (D): upper panel for signal distribution; lower panel for representative blot) for a sample of hiPSC-UF-EV as detected by NTA after separation by SEC; mean and standard deviation (SD) are represented (n=3). Plots show distribution of particle count (E), protein dosage (F), protein surface (G) and luminal (H and I) marker signal (for (G), (H) and (I): upper panel for signal distribution; lower panel for representative blot) for a sample of hiPSC-sEV as detected by NTA after separation by SEC; mean and SD are represented (n=3). Representative TEM images (J) show morphology and size of SEC-hiPSC-UF-EV and SEC-hiPSC-sEV; scale bars are 200nm. Dot plot in (K) shows purity of SEC-hiPSC-UF-EV and SEC-hiPSC-sEV, evaluated as particles to protein ratio; median and interquartile range are represented (n=15 each); statistical analysis was by non-parametric Mann-Whitney test, ****p<0.0001. Dot plot (l) shows purity of hiPSC-sEV and SEC-hiPSC-sEV, evaluated as particles to protein ratio; median and interquartile range are represented (n=25 for hiPSC-sEV; n=15 for SEC-hiPSC-sEV); statistical analysis was by non-parametric Mann-Whitney test, **p<0.01. Abbreviations: BL: baseline; MW:molecular weight; NTA: nanoparticle tracking analysis; prt, particles: sEV, small extracellular vesicles; SEC:size-exclusion chromatography; TEM: transmission electron microscope; hiPSC-sEV: hiPSC-derived sEV; hiPSC-UF-EV: hiPSC-derived ultrafiltration-processed EV.

We further sought to determine hiPSC-sEV integrity and purity after UC isolation. To achieve this, we compared hiPSC-sEV with hiPSC-UF-EV, a process known to impact sEV integrity negatively 53. Both hiPSC-sEV and hiPSC-UF-EV underwent size-exclusion chromatography (SEC), generating 22 distinct fractions (Figure 3A).

hiPSC-UF-EV were separated into particle-enriched (peak at 6-10) and protein-enriched (peak at 14-18) fractions, as confirmed by NTA (Figure 3B) and protein quantification (Figure 3C), respectively. NTA also indicated the presence of particles at low, yet detectable, levels in protein-enriched fractions (12-22). In contrast, particle-enriched fractions showed low protein content, with no consistent concentration peak (from 5 to 8). To verify the identity of the counted particles, western blot analysis was performed. A CD63 signal colocalized with particle-enriched fractions, peaking in fractions 6-8 (Figure 3D). However, protein-enriched fractions consistently exhibited a smeared CD63 signal (from 12 to 16), indicating hiPSC-UF-EV samples contained extraneous EV debris.

hiPSC-sEV underwent a more precise separation using SEC. NTA particle count distribution appeared cleaner, without the persistence of particles in the late fractions (Figure 3E). A particle count peak was detected in fractions 6-7, consistent with protein content, which was consistently more abundant in these particle-associated fractions (Figure 3F). Notably, protein content was negligible or absent in these particle-poor fractions, suggesting that the UC isolation method effectively removed contaminated soluble proteins from hiPSC-sEV, a departure from UF. The particle identities were further assessed, revealing a specific CD63-positive signal tightly concentrated in fractions 6-8 (Figure 3G). Subsequent fractions showed no CD63 signal. The identity and integrity of hiPSC-sEV were confirmed by the FLOT1 (Figure 3H) and ALIX (Figure 3I) evaluation, with both proteins showing strong, distinct signals enriched in fractions 6-8 with no smears. TEM validation underscored the previous observations. The SEC-hiPSC-UF-EV fraction contained more debris and protein aggregates, whereas the SEC-hiPSC-sEV fraction displayed intact EV (Figure 3J).

The particle-to-protein ratio was calculated to assess purity, comparing hiPSC-UF-EV and hiPSC-sEV using SEC. The purity ratio was significantly higher in SEC- hiPSC-sEV than in SEC- hiPSC-UF-EV, with 930 x 10^6^ particles/µg and 0.5 x 10^6^ particles/µg, respectively (Figure 3K). Further analysis demonstrated that SEC improved the purity of hiPSC-sEV. While median values fell within the same 0.1-1 logarithmic range, the purity ratio of SEC- hiPSC-sEV remained significantly higher than that of hiPSC-sEV (Figure 3L).

Collectively, these findings establish SEC as a method to assess and preserve hiPSC-sEV integrity, offering the potential for enhanced hiPSC-sEV purity without the compromise of associated identity markers. Furthermore, we reiterate the detrimental impact of ultrafiltration on hiPSC-EV isolation, endorsing UC as a suitable separation approach.

### Profiling the miRNome cargo of extracellular vesicles

**Figure 4:**
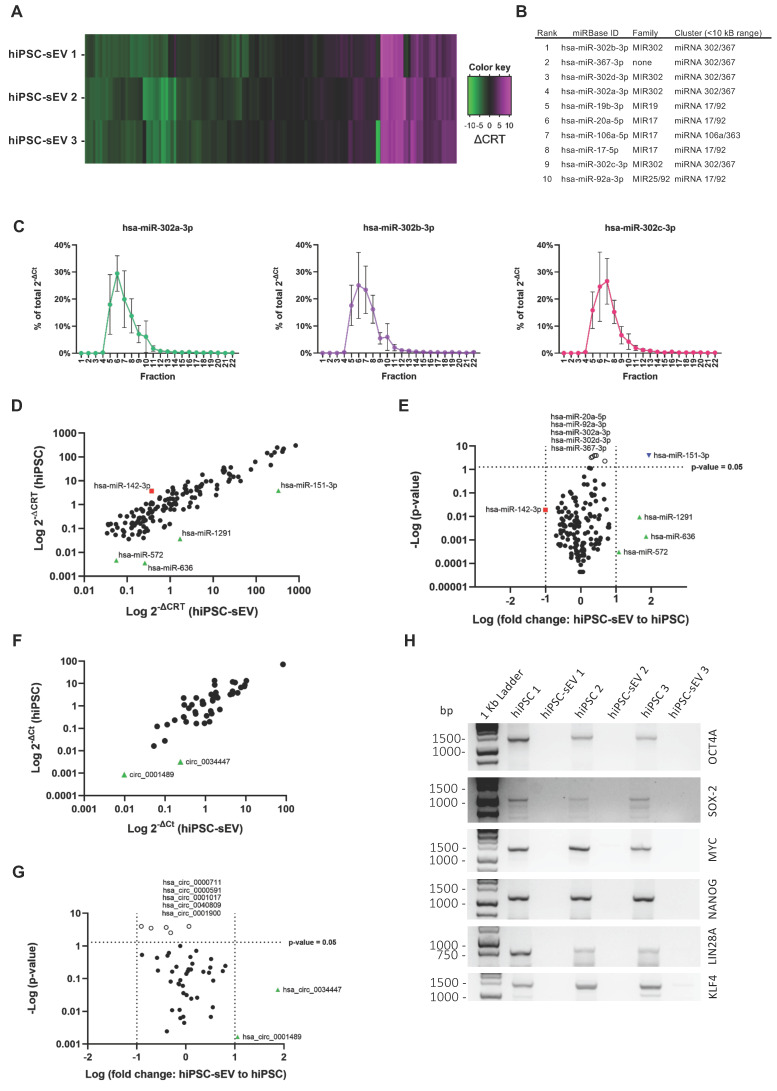
** hiPSC-derived extracellular vesicles shuttle a circRNA and stemness-associated miRNA cargo.** Heatmap in (A) shows miRNome (n=3) of sEV released by hiPSC, reported as differential relative threshold values (∆CRT); differential incorporation into hiPSC-sEV is indicated by the color key. Table (B) lists hiPSC-sEV miRNome top ranked miRNA, indicating family and genomic cluster. Plots in (C) show relative abundance of selected top ranked miRNA for hiPSC-sEV samples, reported as percentage (%) of total 2^-∆Ct^ values determined by qPCR after separation by SEC; mean and standard deviation (SD) are represented (n=3). Scatter plot in (D) shows the comparison between hiPSC (mean of n=3) and hiPSC-sEV (mean of n=3) miRNome in terms of differential expression reported as 2^-∆CRT^ values determined by PCR-array (red square: miRNA underrepresented in hiPSC with a fold change > 1 Log; black dots: miRNA with fold changes within 1 Log; green triangles: miRNA overrepresented in hiPSC-sEV with a fold change > 1 Log). Volcano plot in (E) shows statistical significance of miRNome fold changes of hiPSC-sEV (mean of n=3) to hiPSC (mean of n=3) calculated from PCR-array 2^-∆CRT^ values (red square: miRNA underrepresented in hiPSC with a fold change > 1 Log, but not statistically significant; black dots: miRNA with fold changes within 1 Log and not statistically significant; green triangles: miRNA overrepresented in hiPSC-sEV with a fold change > 1 Log, but not statistically significant; white dots: statistical significant miRNA, but with a fold change < 1 Log; blue inverted triangle: miRNA overrepresented in hiPSC-sEV with a fold change > 1 Log and statistically significant); statistical analysis was by Two-Way ANOVA followed by False Discovery Rate multiple comparisons post-hoc test. Scatter plot in (F) shows the comparison between hiPSC (mean of n=3) and hiPSC-sEV (mean of n=3) selected circRNA panel (n=46) in terms of differential expression reported as 2^-∆Ct^ values determined by qPCR (black dots: circRNA with fold changes within 1 Log; green triangles: circRNA overrepresented in hiPSC-sEV with a fold change > 1 Log). Volcano plot in (G) shows statistical significance of circRNA fold changes of hiPSC-sEV (mean of n=3) to hiPSC (mean of n=3) calculated from qPCR 2^-∆Ct^ values (black dots: circRNA with fold changes within 1 Log and not statistically significant; green triangles: circRNA overrepresented in hiPSC-sEV with a fold change > 1 Log, but not statistically significant; white dots: statistical significant circRNA, but with a fold change < 1 Log); statistical analysis was by Two-Way ANOVA followed by False Discovery Rate multiple comparisons post-hoc test. (H) Agarose gel showing comparison between parental hiPSC and hiPSC-sEV for the amplification of full-length mRNAs of OCT4, SOX2, MYC, NANOG, LIN28A and KLF4. Abbreviations: bp: base pairs; ∆CRT: differential relative threshold; hiPSC: human induced pluripotent stem cells; SEC: size-exclusion chromatography; hiPSC-sEV: hiPSC-derived small extracellular vesicles.

The comprehensive biological characterization of hiPSC-sEV was complemented by the determination of their non-coding (nc) RNA content. First, we employed a high-throughput PCR array method encompassing 754 human miRNAs based on the miRBase version 14 database (https://www.mirbase.org/). This analysis revealed a conserved expression of 147 unique miRNAs across the three distinct hiPSC-sEV batches (Figure 4A and Supplementary Figure 2A). Based on amplification outputs, the average top-ranked miRNAs belonged to pluripotency-associated miRNA families and clusters (Figure 4B and Supplementary Figure 2B). Conversely, the least expressed miRNAs primarily belonged to the MIR515 family, which is associated with human trophoblast differentiation 54,55 (Supplementary Figure 2C). The differential ranking of miRNAs was validated on selected targets using qPCR, which confirmed the same amplification pattern (Supplementary Figure 2D).

To demonstrate that the identified miRNAs were associated with hiPSC-sEV and not influenced by protein contaminants or other particle factors, the top-ranked miRNAs were validated using qPCR in SEC-hiPSC-sEV samples. The results showed clear amplification of all analyzed miRNAs in hiPSC-sEV-enriched SEC fractions 5-8 (Figure 4C). Further analysis focused on comparing the hiPSC-sEV miRNome with that of the parent hiPSC cells, to define specificity in terms of miRNA content. The hiPSC-sEV dataset demonstrated complete overlap with the hiPSC miRNome, underscoring a shared content of the same 147 miRNAs. Upon applying a 10-fold cut-off (sEV-to-hiPSC ratio), the analysis showed the overrepresentation of a few miRNAs in hiPSC-sEV, whereas just one miRNA was underrepresented (Figure 4D), although not significantly different (Figure 4E).

This analysis revealed a distinctive molecular signature of pure hiPSC-sEV, affirming the alignment with the identity of their parent hiPSC.

Finally, we investigated if the miRNA profile of hiPSC-sEV was consistent with previous studies. To this aim, the list of 20 top expressed miRNAs in hiPSC-sEV was compared to other hiPSC-derived EV profiles already published 48-50. The comparison revealed the presence of 50 unique miRNAs and among them only hsa-miR-92a-3p (2%) was common to all lists, belonging to the pluripotency-associated miRNA clusters 17/92. Five miRNAs (10%) resulted common to three out of four profiles and 17 (38%) common to two out of four signatures (Supplementary Figure 2E). The profile most similar to the one described in our work resulted the one published by Bi and colleagues, which shows 11 miRNAs (55%) in common, while the other two profiles were more similar among them.

### Profiling the circRNome cargo of extracellular vesicles

Our analysis into the ncRNA content extended to address the class of circRNA molecules, with the aim to characterize for the first time the circRNA profile of hiPSC-sEV.

A total of 4,747 circRNAs were found to be shared by hiPSC-sEV and hiPSC, presenting a highly similar signal distribution: 98.2% of these molecules exhibited a signal intensity within a log-fold change range (Supplementary Figure 2F).

For a more detailed analysis of the differential expression between hiPSC-sEV and their parental hiPSC, we selected a panel of 46 circRNAs among the molecules detected by microarray for qPCR analysis. Similar to the miRNome cargo, we employed a 10-fold cut-off (sEV-to-hiPSC ratio) and observed an overrepresentation of certain circRNAs within hiPSC-sEV, whereas no circRNAs were found to be underrepresented (Figure 4F). Moreover, these differences lacked statistical significance (Figure 4G).

Given the abundance of pluripotency-associated ncRNA shuttled by hiPSC-sEV, also the presence in sEV of coding full-length mRNA transcripts of the Pluripotency Genes Regulatory Network (PGRN) and other Yamanaka factors 56 was investigated by PCR and detected by gel electrophoresis comparing with the parental hiPSC. The analysis clearly showed the absence of full-length mRNAs of *OCT4*, *SOX2*, *MYC, NANOG, LIN28A* and *KLF4* genes in hiPSC-sEV (Figure 4H).

### Extracellular vesicles elicit a protective response upon acute damage

**Figure 5:**
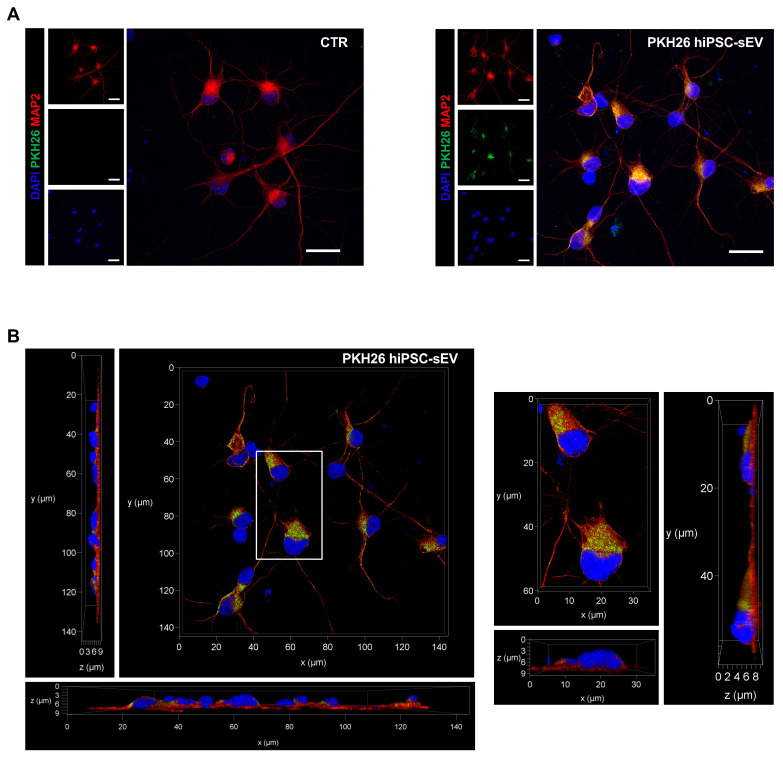
** PKH26-labeled hiPSC-derived extracellular vesicles uptake on neuronal cells.** Confocal images show representative fluorescent signals from DAPI (405, shown in blue), MAP2 (647, shown in red) and PKH26 (561, shown in green) of neuronal cells upon 24h incubation with PKH26-hiPSC-sEV. In (A) are shown two-dimensional projection of single and overlayed signals (objective 63X/1.30 GLYC); scale bars 20 µm. In (B) representative orthogonal views, xy, xz, yz (objective 63X/1.30 GLYC) displaying the intracellular presence of PKH26-hiPSC-EVs. Abbreviations: MAP2: microtubule-associated protein 2; hiPSC-sEV: hiPSC-derived small extracellular vesicles; GLYC: glycerol.

We investigated the ability of hiPSC-sEV to be internalized by neuronal cells for releasing ncRNA cargo into the cytoplasm, thus exerting a biological modulation on injured cells. The uptake was tested using hiPSC-sEV labeled with PKH26 (PKH26-hiPSC-sEV) on human neurons differentiated from NPCs as an *in vitro* model. Fluorescence was detected using confocal microscopy after 24h of PKH26-hiPSC-sEV incubation (Figure 5A). We observed PKH26-positive intracellular particles, as shown in Figure 5A and 5B, demonstrating the successful uptake by the cells. To support these data, using another EV staining and another technique, we confirmed the integration of CFSE- hiPSC-sEV on the same *in vitro* model by flow cytometry, as shown in Supplementary Figure 3A and 3B.

**Figure 6:**
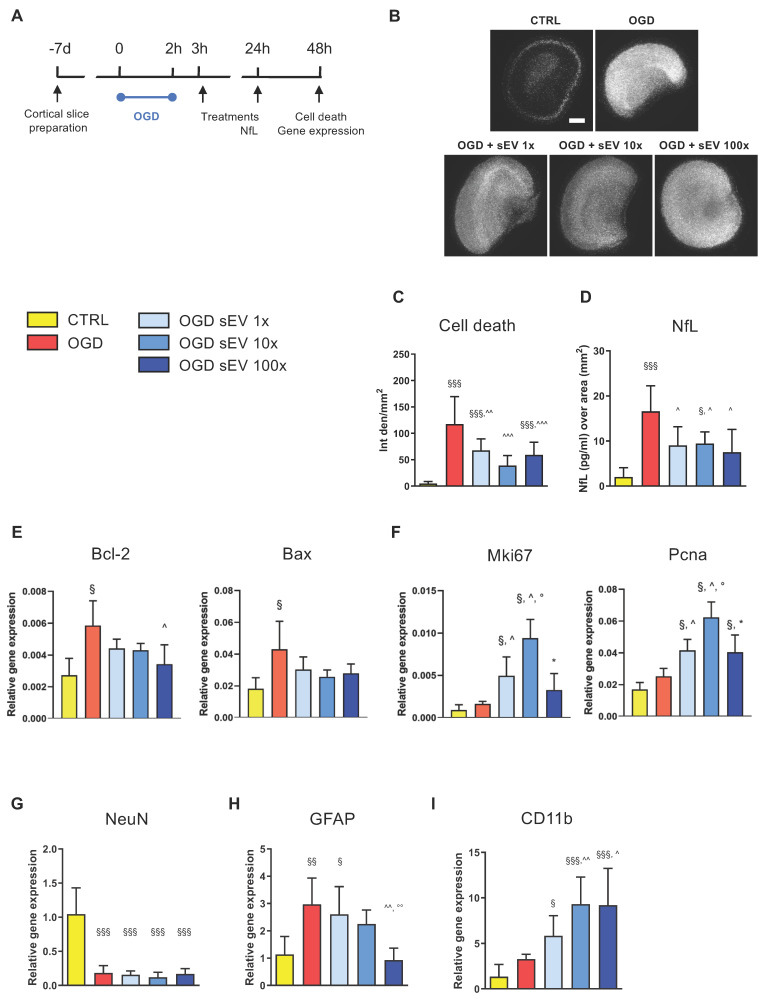
**hiPSC-derived extracellular vesicles exert protective effects after acute brain injury.** (A) Schematic representation of the experimental design used for the assessment of the *ex vivo* model of ischemic damage on organotypic brain slices. (B) Representative images of PI incorporation (bar = 500 μm) and (C) relative quantification. (D) Quantification of NfL release in the culture medium 24h after injury, as index of neuronal damage. (E-I) Real time RT-PCR analysis performed at 48h post-injury of genes involved in cell apoptosis (E, Bcl-2, Bax) and proliferation (F, Mki67, Pcna) or markers of neuronal (G, NeuN), astrocytic (H, GFAP) or microglia (I, CD11b) responses. Data are expressed as mean + SD. Statistical analysis was performed by one way ANOVA, followed by Tukey post-hoc test; §p<0.05, §§p<0.01, §§§p<0.001 vs CTR; ^p<0.05 vs OGD; °p<0.05 vs OGD + sEV 1×; * p<0.05 vs OGD + sEV 10×. Abbreviations: CTR: control; hiPSC: human induced pluripotent stem cells; hiPSC-sEV: hiPSC-derived small extracellular vesicles; NfL: neurofilament light chain; Bcl-2: B cell leukemia/lymphoma 2 protein; Bax: Bcl-2 associated protein x; Mki67: marker of proliferation ki 67; Pcna: proliferating cell nuclear antigen; NeuN: neuron specific nuclear protein; GFAP: glial fibrillary acidic protein; CD11b: cluster of differentiation molecule 11b; OGD: oxygen and glucose deprivation; SD: standard deviation.

Based on these results, we tested the therapeutic potential of hiPSC-sEV in an *ex vivo* model of brain ischemia, represented by organotypic cortical slices subjected to OGD (Figure 6A). A logarithmic dose-response curve was applied, consisting of two subsequent administrations of 0.6-6.0-60.0 × 10^9^ particles/well/administration (1×, 10×, and 100× dose, respectively) at 1h and 24h post-OGD insult. Cell death in brain tissue was evaluated 48h after OGD using a PI incorporation assay (Figure 6B). hiPSC-sEV exhibited a strong protective effect on OGD-injured slices, with a significant reduction in PI incorporation across all applied doses, with the 10x dose showing the highest protection (Figure 6C). Protective effects induced by hiPSC-sEV were confirmed when evaluating NfL, as a proxy of neuronal damage, in the culture media. Compared to OGD untreated condition, all three doses showed a significant reduction of released NfL (Figure 6D).

To investigate the underlying molecular mechanisms of hiPSC-sEV, we assessed the transcript levels of a selected panel of genes related to survival and cell growth. Apoptosis-associated *Bcl-2* and *Bax* were significantly upregulated following OGD, and in particular *Bcl-2* showed a partial rescue upon hiPSC-sEV treatment, with the 100× dose reaching significance (Figure 6E). Proliferation-associated *Mki67* and *Pcna* genes were not altered after OGD, yet exhibited a significant increase upon hiPSC-sEV treatment, compared to control and untreated OGD slices, particularly with the 10× dose (Figure 6F). In order to understand which cell population was associated with proliferative activity, we analyzed the expression of neuronal (NeuN, Figure 6G), astrocytic (GFAP, Figure 6H) and microglial (CD11b, Figure 6I) related genes. The OGD-induced downregulation of NeuN was not affected by hiPSC-sEV treatments. GFAP was upregulated after OGD, and a dose response effect was observed with hiPSC-sEV 100x inducing a significant downregulation. At last, hiPSC-sEV induced an up-regulation of the microglial marker CD11b, with doses 10x and 100x showing the highest effects.

**Figure 7:**
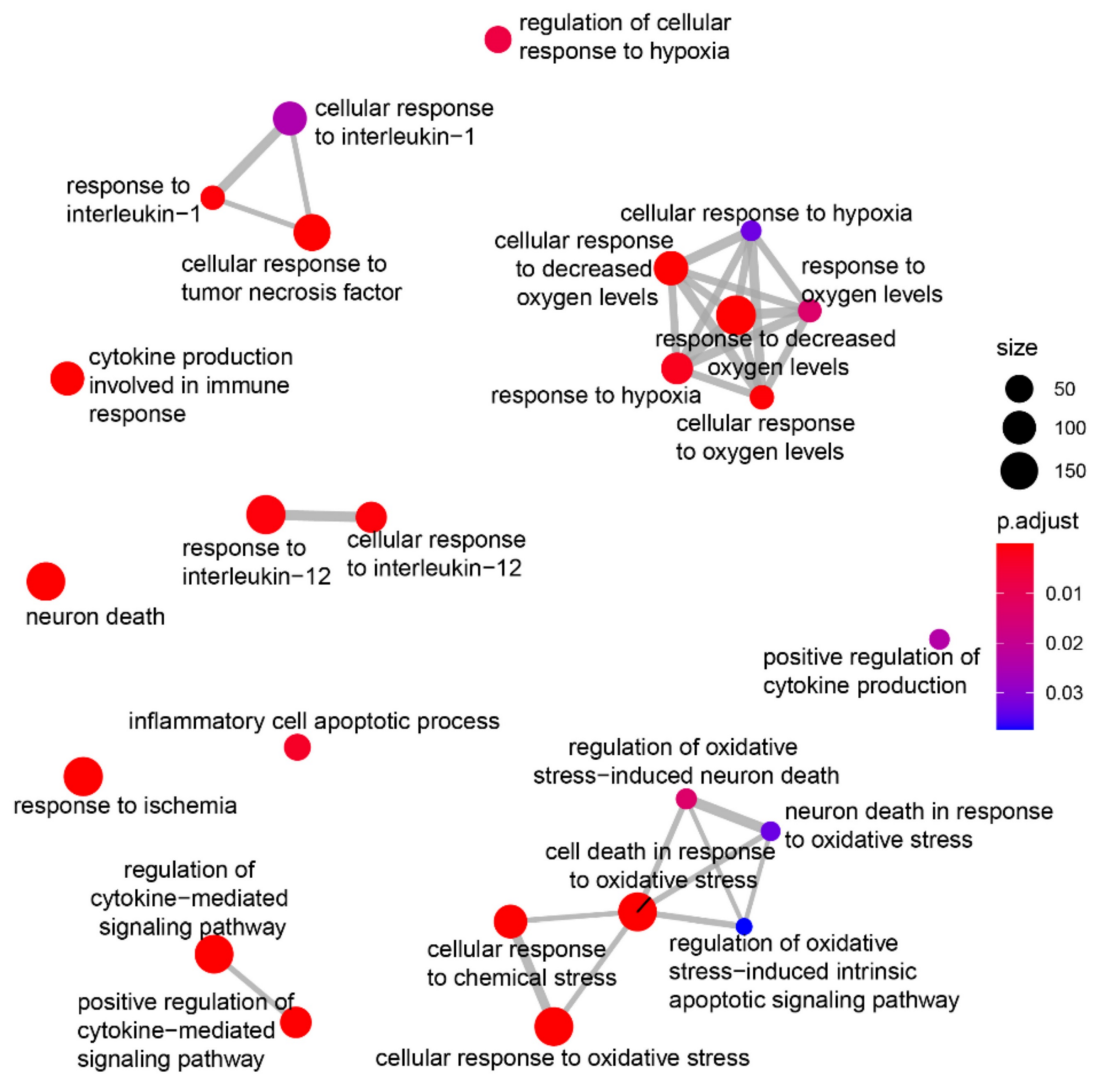
** hiPSC-derived extracellular vesicles circRNA biological role prediction.** Plot showing manual selection of the GO biological processes enriched among the validated targets of the fifteen microRNAs predicted to interact with the most expressed circRNAs and expressed in the frontal lobe. Node size increases according to the number of validated targets related to each term and node color ranges from blue to red according to the adjusted pvalue. Edges thickness represents the percentage of genes in common among the different terms for overlaps greater than 20%.

We then explored the potential role of hiPSC-sEV-shuttled circRNAs in contributing to the observed beneficial effects. The ten most highly expressed circRNAs (Supplementary Figure 3C) were selected based on their normalized array signals. Their potential miRNA targets were predicted using three different algorithms, yielding a list of 269 miRNAs, wherein at least two algorithms coherently predicted a minimum of two binding sites on the same or different circRNAs. Subsequently, 183 miRNAs were found to be expressed in the frontal lobe according to the miRNA tissue Atlas v2.0 40, with 15 exhibiting relevant expression levels, making them potential circRNA targets (Supplementary Figure 3D). Notably, 8 of these miRNAs overlapped with those highly expressed in the microglial cell subtype. To estimate the biological impact of the downregulating these 15 miRNAs, their validated targets were identified using the multiMiR R package 41 and searched for enrichments in disease-related genes and gene ontology biological processes through the DOSE and clusterProfiler packages, respectively 42. This analysis revealed enrichments in hypoxia-related genes, as present in the DISgeNet database, as well as in biological processes involved in hypoxia-related neuronal death and inflammation (Figure 7).

These findings suggest that hiPSC-sEV retain significant and relevant tissue-protective properties for acute neural damage.

## Discussion

The prevailing clinical framework for hiPSC use predominantly focuses on their therapeutic potential within tissue replacement boundaries 57,58. Here, we propose an alternative and possibly complementary approach for hiPSC use based on the release of their EV.

Our group boasts a considerable track record in cord blood research, spanning from oncohematological clinical applications to the unique therapeutic use of MSC 23,52,59-61. We successfully generated hiPSC from this fetal cord blood cell source, starting from cord blood-derived MSC (CBMSC) with the goal of maintaining parental cell young trait and of warranting the safety of these new hiPSC lines 24,62,63.

The introduction of cell-free therapy in the context of regenerative medicine poses both challenges and promises. Innovative therapies, including advanced therapy medicinal products (ATMP) based on EV, necessitate rigorous regulatory considerations. A pivotal aspect involves the precise “identity definition” of the clinical product. Henceforth, we started the work presented herein.

In accordance with MISEV2023 recommendations, our EV underwent thorough characterization based on their protein composition, encompassing selected markers spanning transmembrane, secreted, and cytosolic intracellular-compartments 28. This comprehensive panel of antigens encompassed hiPSC-specific cell membrane markers, immune histocompatibility complexes, hematopoietic and stromal cell-type markers, and organelle-specific molecules. These results significantly expand and advance the current knowledge on hiPSC-sEV.

Flow cytometry and western blot analysis were used to measure tetraspanin protein EV marker levels in hiPSC-sEV and to compare with those in parental cells. The congruence of physical properties and biological attributes ensured accurate hiPSC-sEV identity assessment. Intriguingly, a more in-depth analysis unveiled that hiPSC-sEV presented nucleus-associated markers (i.e., H3 and LMNB1), which could be related to high nucleus-to-cytoplasm ratio typical of hiPSC, potentially facilitating nuclear material in exosome biogenesis. Notably, no other organelle-associated markers (i.e., Golgi apparatus, endoplasmic reticulum, mitochondria, cytoskeleton, lysosomes) were found, confirming compliance with MISEV2023 standards and confirming the absence of apoptotic bodies in hiPSC-sEV preparations. Furthermore, consistent with previous reports, we showed that hiPSC-sEV were negative for hematopoietic markers (CD45), but positive for integrins 64,65, EV-associated markers 48,64-66, and pluripotency-associated antigens (SSEA4) 64,65.

To validate the physical properties of hiPSC-EV, we applied analytical methodologies to obtain a clear indication that hiPSC-EV were enriched in small EV.

To envision the large-scale standardized manufacturing processes required for possible future clinical applications, we assessed the kinetics of hiPSC-sEV production. We confirmed that they possess floating properties and a density compatible with EV identity, compared to similar ranges defined for other cell sources 67-71.

Furthermore, we employed a chromatographic technique to thoroughly pinpoint the identity and biological content of hiPSC-sEV. This technique allowed for the precise association of selected biomolecules with hiPSC-sEV and the assessment of their integrity and purity. The application of size-exclusion chromatography significantly improved the particle-to-protein ratio compared to ultracentrifugation or ultrafiltration-processed EV, all while retaining EV markers, miRNA content, and proper morphology.

An essential requirement for the therapeutic application of hiPSC-sEV is their ability to interface with or be internalized by target cells, thereby triggering their effects 72 or transferring the bioactive cargo within the EV lumen to modulate intracellular molecular pathways 73. Uptake of hiPSC-sEV has been demonstrated in several cell types, such as endothelial cells 74,75 and hepatic stellate cells 48. We successfully demonstrated the uptake of hiPSC-sEV by human neurons.

Still in the context of possible future clinical use, to demonstrate that hiPSC-sEV released into the extracellular environment and taken by neighbouring cells cannot lead their conversion into hiPSC by providing the necessary factors for inducing pluripotency, in this study we determined that hiPSC-sEV did not carry full transcripts of genes involved in their reprogramming process.

To test the therapeutic potential of hiPSC-sEV, we used an *ex vivo* model of acute brain damage. The short treatment window for acute damage and the complex multifactorial inflammatory cascade surrounding it underscore the advantages of EV-based therapeutics over cell-based therapies. Ideally, EV therapeutics could be developed as ready-to-use off-the-shelf drugs, easily available to physicians operating under urgent needs. In our *ex vivo* model of ischemic brain injury, we observed a consistent reduction in OGD-induced cell death and neuronal damage obtained with all three doses tested recapitulating what has previously been observed using the secretome derived from human amniotic MSC or from human umbilical cord perivascular cells 34,35. In view of identifying a solid biomarker able to monitor neuronal damages and the efficacies of therapies, we employed the use of NfL 76. This biomarker reflects the structural integrity of neurons in human brains and it is translationally valid, supported by its prognostic value after acute brain injury 77-80 and its adoption as a primary outcome measure in Phase II trials 81. Establishing its preclinical validity as a pharmacodynamic biomarker will enhance the translation of neuroprotective treatments from lab to clinical settings 82. In our experimental setting, NfL was nicely and statistically reduced after hiPSC-sEV treatment in comparison with the ODG levels, confirming the pharmacodynamic validity of NfL biomarker for acute brain injury 35. We further analyzed the hiPSC-sEV-induced effects on injured brain tissue at gene expression levels finding an induction of proliferation-associated genes. No treatment effects on neuronal gene was observed, thus indicating a neuroprotective more than a regenerative mechanism of action of hiPSC-sEV. Instead, clear hiPSC-sEV dose effects were found on glial cells, with reduction of astrocyte and induction of microglia activation. These results are in agreement with previous work from our group in the *in vitro* model 34,35 and suggest the role of microglia activation in the observed protection 83,84.

Similar results in a different inflammatory context, in a diabetic mouse model, were obtained by Levy *et al.* starting from EV isolated from a hiPSC line derived from bone marrow CD34+ cells obtained from a healthy 31-year-old donor 85. And again, using a commercially available hiPSCs, Saneh *et al.* showed that hiPSC-EV attenuated hyperoxic injury in a fetal murine lung explant model 86.

On the whole, our data suggest that sEV exert a protective effect on brain tissue exposed to ischemic conditions and modulate astroglial and microglia reactions. However, additional experiments are necessary to confirm that sEV support neuronal survival and activity and to unveil the underlying mechanisms.

Indeed, several mechanisms of action have been proposed for the effect of EV in regenerative medicine, including mitochondrial transfer 87,88 and RNA 7,89-91 and protein 86,92-94 delivery; however, a defined and shared consensus is still missing. The transfer and direct action of specific miRNAs 95-101 has also been proposed as a mechanism of action for EV. Several studies have supported this hypothesis, since various miRNAs specifically involved in inflammatory processes have been found to be abundant in EV 102-105. However, there are still some concerns on whether miRNA transfer from EV to target cells can exert therapeutic effects 106-108. Nonetheless, the miRNA cargo of our hiPSC-sEV could potentially affect inflammatory signaling processes, which could be attributed to the presence of miRNA subset targeting anti-inflammatory mRNAs, namely, the hsa-miR-24-3p 109 and hsa-miR-130a-3p 110,111.

Our hiPSC-sEV revealed similarities and discrepancies with miRNA profiles showed by other groups that may reflect the differences in hiPSC lines employed as EV source and hiPSC-EV isolation methods. In addition, the method adopted to investigate miRNA profile can influence the results. Indeed, likewise our work, Bi et colleagues studied EV secreted from hiPSC obtained via a non-integrative reprogramming method, starting from MSC, while the other groups both used hiPSC derived from fibroblasts via an integrative method. Moreover, Bi *et al.* followed an isolation workflow consistent with our protocol and investigated miRNA profile via a miRNA microarray. On the other side, the other two groups adopted different methods for both EV isolation and miRNA sequencing.

Among these data sets, some differences were reported that could be due to the cells of origin, to the reprogramming methods employed to obtain hiPSC and to the protocols adopted to isolate hiPSC-EV populations and to analyze their miRNA cargo.

The ncRNA family has emerged as a key player in regulating molecular networks associated with differentiation pathways 112-116. Among ncRNAs, circRNAs have recently gained attention as novel regulators of physiological cell functions 117-121. Although initially perceived as mere byproducts of mRNA splicing 122-126, recent studies unveiled a plethora of endogenous circRNAs across various tissues and organisms under diverse conditions, highlighting their pivotal roles in cellular biology and pathophysiology 127,128. As EV are considered a promising drug and potential delivery vectors, EV carrying circRNAs hold promise for treating pathologic conditions 129. Herein, we contribute to the largest hiPSC-EV circRNome catalog, shedding light on their possible role in the field of functional ncRNAs. This groundbreaking study introduces a network of interactions between mRNAs, miRNAs, and circRNAs within hiPSC-EV, suggesting circRNAs' involvement in the anti-inflammatory effects observed with EVs.

circRNAs have a stable structure, the ability to resist RNA enzymes, and sequence-conserved characteristics. Their regulatory role in injury and regeneration might be favored 130-135, thus laying a foundation for their future clinical application. Recent innovative research has presented EV-circRNAs as potential players in the ischemic injury processes 136,137. Cellular stresses like hypoxia and inflammation, associated with several pathological conditions, including cerebral ischemic injury, significantly impact the regulation of circRNAs 138-140. Although the precise role of EV-circRNAs in pathophysiological settings remains unclear, a recent study demonstrated the potential of engineered EV for delivering candidate circRNAs, which led to the restoration of a specific circRNA (circSCMH1) levels in rodent and non-human primate ischemic stroke models, hinting at the therapeutic viability of EV-circRNA strategies 141. In this work, we defined the largest hiPSC-EV circRNome ever reported as a possible novel actor in the area of non-coding functional RNAs. Although our study sheds light on this possible role in injury and regeneration, it is essential to recognize that the intricate mechanisms underlying EV-based therapeutics likely comprise multifactorial and interconnected pathways, culminating in complex and complementary biological cargo responses.

## Conclusions

Our study introduces a compelling avenue for the near-term clinical application of hiPSC-derived extracellular vesicles in the field of cell-free therapy. This approach has the potential to revolutionize regenerative medicine by harnessing the inherent reparative capabilities of EV, thereby promising a future rich in therapeutic possibilities. As the field advances, further investigations into the precise mechanisms underpinning the diverse therapeutic effects of EV will unveil the full extent of their potential impact.

## Supplementary Material

Supplementary figures.

Supplementary data.

## Figures and Tables

**Table 1:** Primary antibodies employed in western blot and blotting strategies.

Target	Species reactivity	Vendor	Cat. number	Dilution	Transfer Stack
ACTB	Human	Sigma-Aldrich	A5441	1:5 000	Nitrocellulose
ALIX	Human	Santa Cruz Biotechnology	sc-53538	1:500	Nitrocellulose
ANXA1	Human	BD Biosciences	610066	1:5 000	Nitrocellulose
CALR	Human	BD Biosciences	612136	1:250	Nitrocellulose
CD62E	Human	ThermoFischer Scientific	14062782	1:100	Nitrocellulose
CD63	Human	Millipore	CBL553	1:100	Nitrocellulose
CD81	Human	BD Biosciences	555675	1:250	Nitrocellulose
CD9	Human	BD Biosciences	555370	1:500	Nitrocellulose
FLOT1	Human	BD Biosciences	610820	1:500	Nitrocellulose
FLOT2	Human	BD Biosciences	610383	1:250	Nitrocellulose
FN	Human	BD Biosciences	610077	1:5 000	PVDF
GAPDH	Human	Santa Cruz Biotechnology	sc-47724	1:200	Nitrocellulose
GM130	Human	Santa Cruz Biotechnology	sc-55591	1:500	Nitrocellulose
H3	Human	Cell Signaling Technologies	4499S	1:2 000	Nitrocellulose
HSP70	Human	BD Biosciences	610607	1:250	Nitrocellulose
LAMB2	Human	BD Biosciences	610722	1:250	PVDF
LMNB1	Human	Santa Cruz Biotechnology	sc-374015	1:250	PVDF
SSEA-4	Human	BD Biosciences	560073	1:500	Nitrocellulose
TRA1-60	Human	Abcam	ab16288	1:250	PVDF
TRA1-81	Human	Abcam	ab16289	1:250	PVDF
UQCRC1	Human	Abcam	ab110252	1:1 000	Nitrocellulose

**Table 2:** Secondary antibodies employed in western blot. HRP: Horseradish peroxidase linked: IgG (H+L): Gamma Immunoglobulins Heavy and Light chains

Target	Host	Conjugate	Immunogen	Vendor	Cat. number	Dilution
Rabbit	Donkey	HRP	IgG (H+L)	GE Healthcare	NA934 1ML	1:3 000
Mouse	Goat	HRP	IgG (H+L)	BioRad	1706516	1:3 000

**Table 3:** Primers employed in qPCR.

Target	Specie	Forward primer				Reverse primer
circ_0006789	Human	5'-TCCTTTCCCTTTGAGACCGT-3'				5'-GAGAGAGAACTGATCTCGGGGT-3'
circ_0001489	Human	5'-CTCTAGGCTTGTTAGTGGGTT-3'				5'-CAGGGTGCTTAGGGAGCATA-3'
circ_0012634	Human	5'-GAAATTCACAAGCGCACAGGA-3'				5'-TGCGGAGTCCATCATGTCAC-3'
circ_0092283	Human	5'-CAAGACTCTGGACCCCAAGG-3'				5'-AGAGCCCAGAGTGGGAGAAG-3'
circ_0080210	Human	5'-TCACGCCGGGTTCTTTACCT-3'				5'-GCTCACCCACATCTACCACTTA-3'
circ_0001360	Human	5'-TCGTCGTCATCGTCATCTTC-3'				5'-GGGTAATACTGCCGCTGGTA-3'
circ_0001973	Human	5'-CACAGACACAGAGTGAGAAGCA-3'				5'-CATGATGGTGACACTGGATGC-3'
circ_0008234	Human	5'-AAAGGGAAAGGTTCCCGTGT-3'				5'-GCTGCTGCTGGAGGAGAAC-3'
circ_0008253	Human	5'-GCCTGCTCTCAGTTTGTTCC-3'				5'-TTCCGAGGATACCTCTGGTC-3'
circ_0040809	Human	5'-GATCTGGTCACGAACAAGCA-3'				5'-CCGGTCAACACGAAAGAGTT-3'
circ_0007001	Human	5'-TTTTCATGAACGTGGACAGC-3'				5'-CGCTGGCGAATACTGTCTCT-3'
circ_0000247	Human	5'-AGGGAGAGTGTTTTCCTGCTC-3'				5'-CTGGCATGGTACATGGAGAG-3'
circ_0000682	Human	5'-ACAGGGACGTCCTCATTGTC-3'				5'-GTCACATTTCATCCCCTGGT-3'
circ_0015232	Human	5'-TCAGCCTCACCTTCAAGGAG-3'				5'-GTTGGGCAGGGGCACATTAT-3'
circ_0023919	Human	5'-GCCCAATGATCTGCTTGATT-3'				5'-AGTGTAGTTGCCCTGCTTGC-3'
circ_0008432	Human	5'-GGGCCATGAAGGATGAGGAG-3'				5'-TTGAGGGCGGCCACATC-3'
circ_0034398	Human	5'-ATGCGCCCTCATTAATGGCT-3'				5'-ATGTGTTTCTGGTACTCCTGGG-3'
circ_0006566	Human	5'-ACGAGATCTGCCCTCCTTG-3'				5'-AAGTATCCTAAAGGGCCGTCA-3'
circ_0001009	Human	5'-TACCTCCTCCTCCCCAGTTC-3'				5'-TGTTCTCAGCTGCCAACTACA-3'
circ_0049462	Human	5'-CGATGGTGTTTGTGACTGCT-3'				5'-GGGGCTTATAGCCAGTGTTG-3'
circ_0003249	Human	5'-ATCATTCCGCCTTTTGGGGA-3'				5'-TCTAGAACCACCCCGTCTGT-3'
circ_0003205	Human	5'-AACCGGGTAACAGCAGAGAG-3'				5'-GCAGCCAAAAGACAACAGGT-3'
circ_0085173	Human	5'-GCGCCTATCTCAAAGACGAC-3'				5'-GGGAAAGGTTCACTGGAACA-3'
circ_0000591	Human	5'-AAAACGAGACTTTCTTGGTTTCA-3'				5'-CTGCTGTTTCTCCTCCATGA-3'
circ_0001324	Human	5'-TCGTTTTCCAACCCCTTCTCC-3'				5'-TAGCTGATTGGTGGGCTGTT-3'
circ_0061774	Human	5'-GGGCTTCTACGTCATCTTCG-3'				5'-TATGTAGGAGTGCGGGGTTC-3'
circ_0003472	Human	5'-GACGTTTCACTGCTGCTGAG-3'				5'-CCAATTGGAAGGAACAGAGC-3'
circ_0001136	Human	5'-TGCCTCTATGACCTGCAGAA-3'				5'-TATAAACTGCCTGGCCGAAT-3'
circ_0000437	Human	5'-AGGGTCATAGAAAGGCAGCA-3'				5'-ATGGGTTACATGCCCAAGAG-3'
circ_0005035	Human	5'-AGCCGATGTGTGAGAAGACC-3'				5'-GATGAGCAGGATGTGGAGGT-3'
circ_0006413	Human	5'-TGGACCGTATTCTCCAAATAGC-3'				5'-GTCCAACAGATGAGGCTGCT-3'
circ_0000002	Human	5'-CCGTCTTCTCCATGATCCAG-3'				5'-CATAGCGAGAAGGAGGTTGC-3'
circ_0000921	Human	5'-TTTTACTGGGGGACAACTGG-3'				5'-GGCAAGGTGCTGAGTCTTTC-3'
circ_0034447	Human	5'-CTCCTGTGATGAGCTGTCCA-3'				5'-CCATTCACCACGTTGTTGTC-3'
circ_0008348	Human	5'-TTCAAGAACGACCCCTACCA-3'				5'-GGTCACAGCGGAAGCACTC-3'
circ_0000818	Human	5'-GCTGAGTTCCTGGACTGGAG-3'				5'-GCCAGATGTACAAGGGAAGC-3'
circ_0000711	Human	5'-AACTCATCATCGAGCCCATT-3'				5'-TGGTAAGCAAAGTGGTGTGG-3'
circ_0001741	Human	5'-CGGCGCACAGAAATTATAGA-3'				5'-CATGGTCTGTGCAGCAAAAT-3'
circ_0001436	Human	5'-TCCAACACTTCAGCCTGGTT-3'				5'-CTCCTTCCAGGGCATCATAA-3'
circ_0004338	Human	5'-TGGTGGTTCGAGAATGTCAA-3'				5'-TGTGCTCCTGCTCATACTGG-3'
circ_0007334	Human	5'-AGGCAAAGAGTTGGCACACTA-3'				5'-TGGGCCTTTATCATCTTGCACTT-3'
circ_0001663	Human	5'-GCTCACCTTGGCTACCTGAA-3'				5'-TCAACAACACATGTCAGCCATA-3'
circ_0001017	Human	5'-TTGGAAAATGTGATAAAAACAAGG-3'				5'-CTGAAATCAAGCAGCTGACG-3'
circ_0001821	Human	5'-TTGGGTCTCCCTATGGAATG-3'				5'-CATCTTGAGGGGCATCTTTT-3'
circ_0001900	Human	5'-TGTGCTCCTGCTCATACTGG-3'				5'-ACGTTCAGTGCCTCGAAAGA-3'
circ_0073244	Human	5'-GGACAAGCAAGGCAAAGTGA-3'				5'-TCCTCTTGGCTCCTTGGGTAA-3'
miR124a-5p	Human	5'-AGGCACGCGGTGA-3'				miScript Universal Primer
miR302a-3p	Human	5'-GCAGTAAGTGCTTCCATGT-3'				miScript Universal Primer
miR302b-3p	Human	Hs_miR-302b_1, MS00003906				miScript Universal Primer
miR302c-3p	Human	5'-AGTAAGTGCTTCCATGTTT-3'				miScript Universal Primer
miR500a-5p	Human	5'-GTAATCCTTGCTACCTGGGT-3'				miScript Universal Primer
miR597-5p	Human	5'-GTGTCACTCGATGACCAC-3'				miScript Universal Primer
*Bcl-2*	Murine	5'-GTGCCTGTGGTCATGGATCTG-3'				5'-CCTGTGCCACTTGCTCTTTAG-3'
*Bax*	Murine	5'-GAGAGGCAGCGGCAGTGAT-3'				5'-TGCTCGATCCTGGATGAAACC-3'
*Gapdh*	Murine		5'-GCAGTGGCAAAGTGGAGATTGT-3'			5'-CGTTGAATTTGCCGTGAGTGGA-3'
*Mki67*	Murine		5'-GATAACGCCACCGAGGACAA-3'			5'-ATGGATGCTCTCTTCGCAGG-3'
*Pcna*	Murine	5'-ACCTTTGAAGATTGCTCCTGAGA-3'				5'-ACTTGGTGACAGAAAAGACCTCA-3'
*NeuN*	Murine	5'-CAGACGGTGCCGCAGG-3'				5'-ATGTAGTCGTTTGGGCTGCT-3'
*GFAP*	Murine	5'-GAAAACCGCATCACCATTCC-3'				5'-TCGGATCTGGAGGTTGGAGA-3'
*CD11b*	Murine	5'-GAGCAGCACTGAGATCCTGTTTAA-3'				5'-ATACGACTCCTGCCCTGGAA-3'
*ActB*	Murine	5'-GCCCTGAGGCTCTTTTCCAG-3'				5'-TGCCACAGGATTCCATACCC-3'
*KLF4*	Human	5'-CAGCCACCTGGCGAGTCT-3'				5'-GTAAGGCGAGGTGGTCCG-3'
*LIN28A*	Human	5'-CCTTTGCCTTCGGACTT-3'				5'- CCTGATAGCAAAAGAATA -3'
*MYC*	Human			5'-ATGCCCCTCAACGTTAGCTTCA-3'		5'-TTACGCACAAGAGTTCCGTAGCTG-3'
*NANOG*	Human	5'-CTGGAGGTCCTATTTCTCTA -3'				5'-AAAAATCCTATGAGGGATGG-3'
*OCT4*	Human				5'-GGTTGAGTAGTCCCTTCG-3'	5'-CTTAATCCCAAAAACCCTGG-3'
*SOX2*	Human				5'-AACATGATGGAGACGGA-3'	5'-TTTCTTTGAAAATTTCTCCCC-3'
						

## Abbreviations

EV: extracellular vesicles
hiPSC: human induced pluripotent stem cells
MSC: mesenchymal stromal cells
sEV: small extracellular vesicles
cGMP: current good manufacturing practice
OGD: oxygen and glucose deprivation
NTA: nanoparticle tracking analysis
CBMSC: cord blood-derived MSC
ATMP: advanced therapy medicinal products
SDG: sucrose density gradient
TEM: transmission electron microscope
SEC: size-exclusion chromatography
NfL: neurofilament light chain
NPC: neural progenitor cells
nc-RNA: non-coding RNA
PGRN: pluripotency genes regulatory network
