# Supplementary material for: Extracellular Vesicles from Human Induced Pluripotent Stem Cells Exhibit a Unique MicroRNA and CircRNA Signature
Source: Int J Biol Sci. 2024 Nov 22;20(15):6255–78. doi: 10.7150/ijbs.100113 (PMC11628337; doi:10.7150/ijbs.100113)
Supplement: Supplementary file 1 — Supplementary figures. [file ijbsv20p6255s1.pdf]

Supplementary figure 1. Human induced pluripotent stem cells release small extracellular vesicles. Image (A) is a plot generated by NanoSight NS300 software and shows an overlay of size distribution of sEV (n=3) released by hiPSC. Plot (B) shows linear regression result of SDG experiments (n=3). Representative TEM image (C) shows morphology and size of SDG-purified hiPSC-sEV; scale bar is 100 nm. Blots in (D) show western analysis comparisons between parental hiPSC and released sEV (n=2) for surface antigens.

Abbreviation list: hiPSC-sEV: hiPSC-derived small extracellular vesicles; hiPSC: human induced pluripotent stem cells; nD20: refractive index temperature compensated; SDG: sucrose density gradient; TEM: transmission electron microscopy.

Supplementary figure 2. Supplementary characterization of hiPSC-sEV surface markers and miRNA/circRNA cargo. Scatter plots in (A) show miRNome comparison between different hiPSC-sEV batches in terms of differential expression, reported as $2^{-\Delta CRT}$ values determined by PCR-array. Graphs (B) show miR-302/367 (upper panel), miR17/92 (middle panel) and miR106a/363 (bottom panel) clusters host genes. Table (C) lists hiPSC-sEV miRNome bottom ranked miRNA, indicating family and genomic cluster. Histogram (D) shows differential expression, reported as $2^{-\Delta Ct}$ values determined by qPCR, of a selected panel of top (n=3, white bars) and bottom (n=3, black bars) ranked hiPSC-sEV miRNA; mean and standard deviation are represented. (E) Venn diagram representation of the hiPSC-EV miRNA profiles described in this paper and in Povero et al, Bi et al, Louro et al. Violin plot in (F) show signal distribution of circRNAs detected by micro-array comparing hiPSC-sEV with hiPSC.

Abbreviation list: AU: arbitrary units; bp: base pair; hiPSC: human induced pluripotent stem cells; Kb: kilobases; prt: particles; hiPSC-sEV: hiPSC-derived small extracellular vesicles.

Supplementary figure 3. Cellular uptake of hiPSC-sEV and the candidates as cargo responsible of hiPSC-sEV therapeutic effect. (A-B) Dot plots and histogram show representative flow cytometry analysis of control and incubated with CFSE+ hiPSCs-EV (n=2) neuron specimens (CFSE indicated as FITC). Table (C) shows the top 10 EVs expressed circRNAs identifiers together with their transcript annotation. Table (D) summarizes the 15 miRNAs predicted to target those circRNAs and expressed at relevant levels in frontal lobe. Abbreviation list: hiPSC-sEV: hiPSC-derived small extracellular vesicles.

(S1)

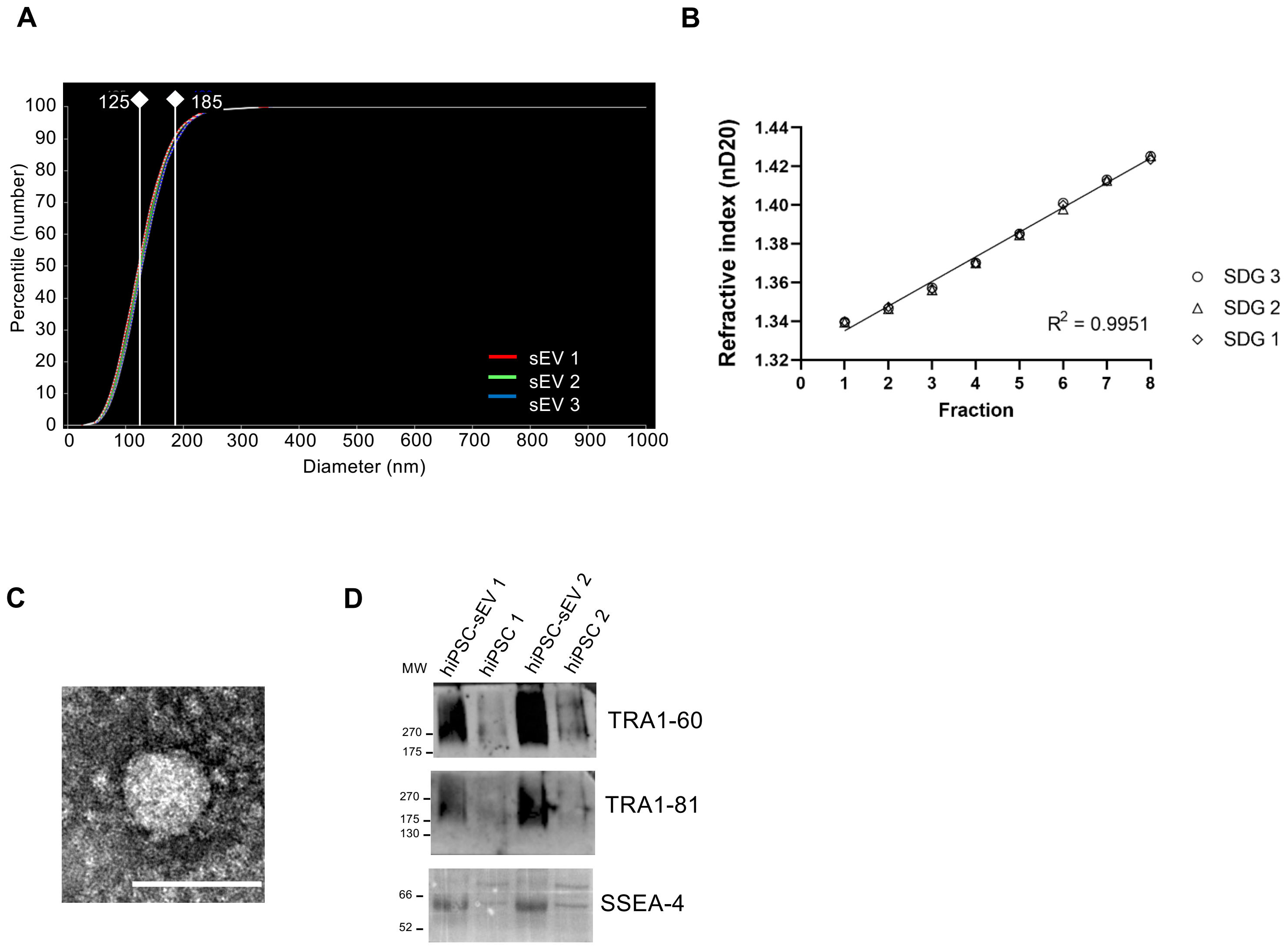
A
125
185
Percentile (number)
Diameter (nm)
sEV 1
sEV 2
sEV 3
B
Refractive index (nD20)
Fraction
R^2 = 0.9951
SDG 3
SDG 2
SDG 1
C
D
MW
hiPSC-sEV 1
hiPSC 1
hiPSC-sEV 2
hiPSC 2
TRA1-60
TRA1-81
SSEA-4
270
175
130
66
52

(S2)

A

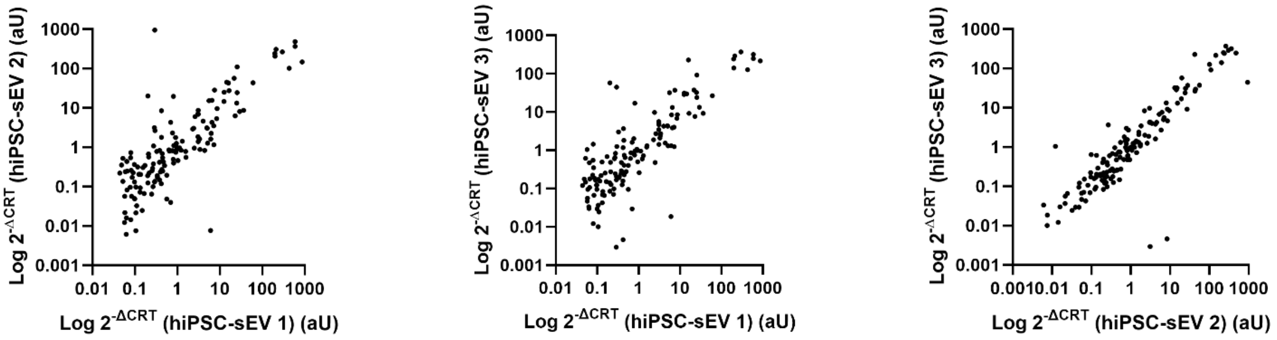

B

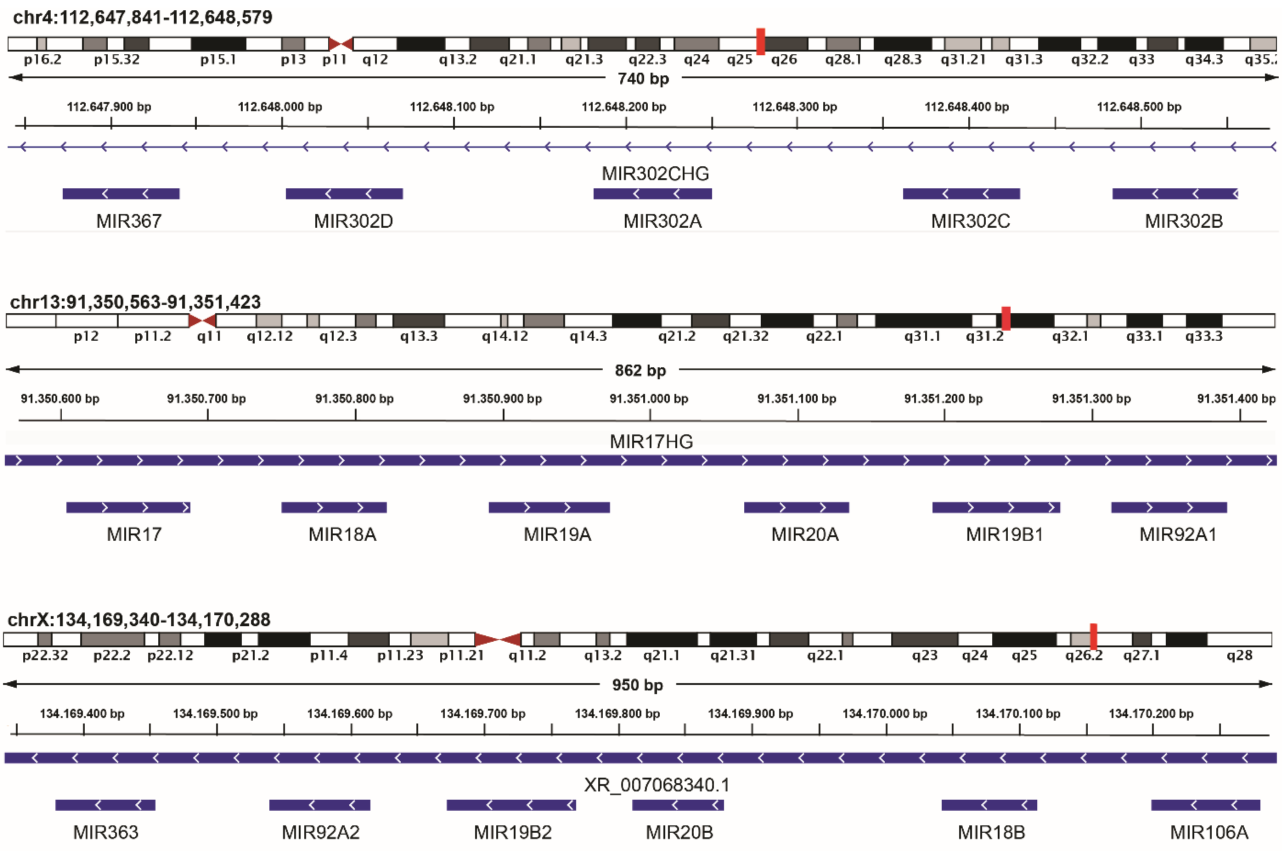

C

Rank	miRBase ID	Family	Cluster (<10 kB range)
144	hsa-miR-526b-5p	MIR515	miRNA 512-2/518b
145	hsa-miR-500a-5p	MIR500	miRNA 532/502
146	hsa-miR-455-5p	none	none
147	hsa-miR-572	none	none
148	hsa-miR-512-5p	MIR506	miRNA 512-1/520e
149	hsa-miR-526a-5p	MIR515	miRNA 525/519d
150	hsa-miR-15a-5p	MIR15/16	miRNA 15a/16-1
151	hsa-miR-597-5p	None	none
152	hsa-miR-520e-3p	MIR515	miRNA 512-1/515-2
153	hsa-miR-29b-3p	MIR29	miRNA 29b-1/29a

D

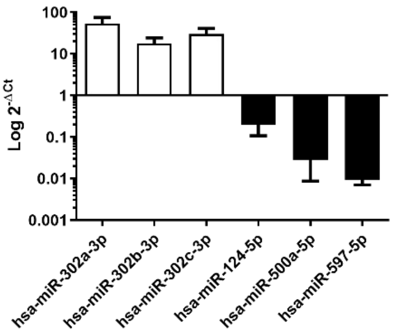

E

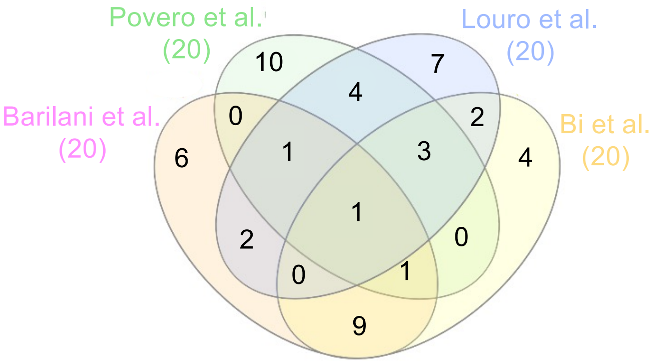

F

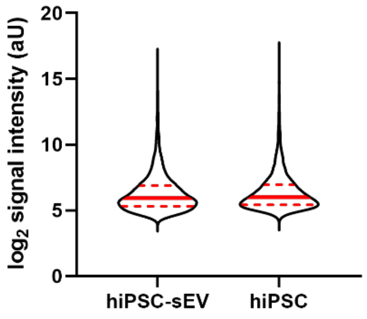

(S3)

A

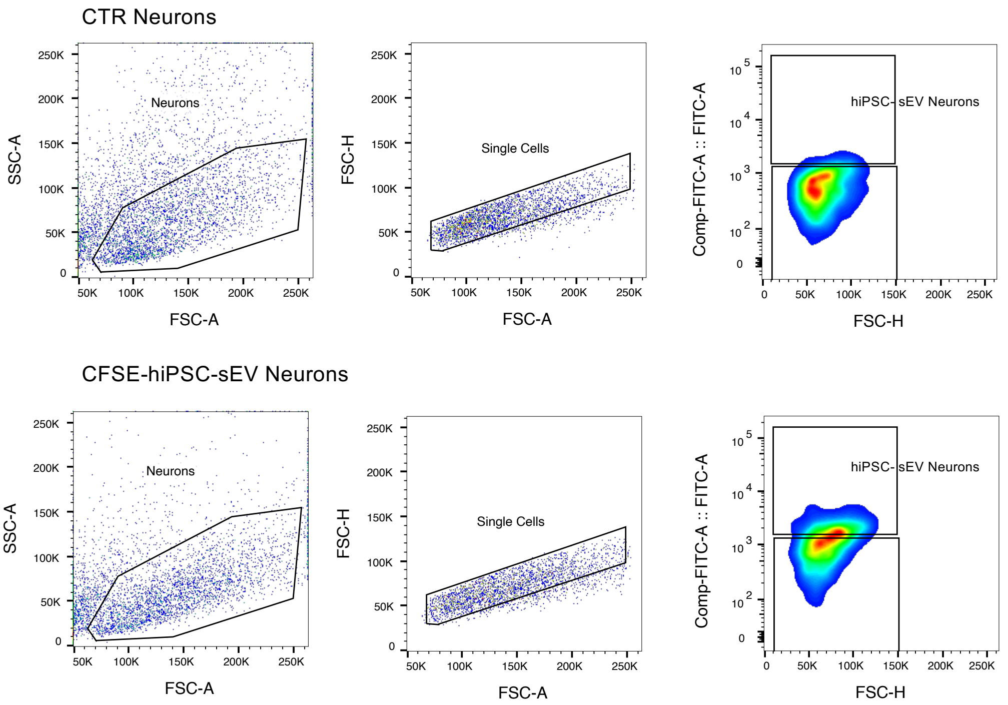

B

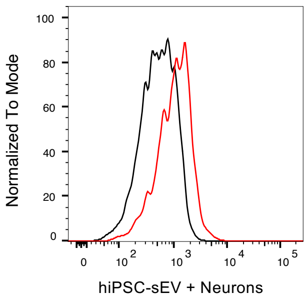

	Sample Name	Subset Name	Count	Median: Comp-FITC-A
☐	CTR Neurons	Single Cells	3733	537
☐	CFSE-hiPSC-sEV Neurons	Single Cells	2322	1102

C

Top sEV circRNA array ID	Alias	Chr	txStart	txEnd	GeneSymbol	Strand
hsa_circRNA_100375	hsa_circ_0006758	chr1	165859440	165860559	UCK2	+
hsa_circRNA_105027	hsa_circ_0006789	chrX	118544152	118544325	SLC25A43	+
hsa_circRNA_001506	hsa_circ_0000981	chr2	20240809	20240905	LAPTM4A	-
hsa_circRNA_102890	hsa_circ_0002867	chr2	200245086	200298237	SATB2	-
hsa_circRNA_000629	hsa_circ_0000775	chr17	43012303	43012398	KIF18B	-
hsa_circRNA_103188	hsa_circ_0062682	chr22	26936754	26937684	TPST2	-
hsa_circRNA_000956	hsa_circ_0001489	chr5	59770958	59771235	PDE4D	+
hsa_circRNA_000274	hsa_circ_0000919	chr19	19760861	19761115	ATP13A1	-
hsa_circRNA_104017	hsa_circ_0004004	chr5	172359438	172362313	ERGIC1	+
hsa_circRNA_102782	hsa_circ_0055630	chr2	96905450	96906426	LOC285033	+

D

miR	n binding sites	frontal_lobe expr
hsa-miR-9-5p	2	24722.08764
hsa-miR-138-5p	2	4129.123776
hsa-miR-24-3p	2	3908.75962
hsa-miR-708-5p	2	827.6093796
hsa-miR-744-5p	2	712.4819363
hsa-miR-330-5p	3	182.9692156
hsa-miR-29b-2-5p	2	121.9343803
hsa-miR-214-3p	2	70.64043719
hsa-miR-1179	2	55.93868428
hsa-miR-4516	2	53.08355833
hsa-miR-30c-1-3p	3	24.88092356
hsa-miR-30c-2-3p	3	14.12116305
hsa-miR-1908-5p	2	11.15587018
hsa-miR-1226-3p	2	10.88440215
hsa-miR-431-3p	2	10.42536501
